# Dilute concentrations of maritime fuel can modify sediment reworking activity of high‐latitude marine invertebrates

**DOI:** 10.1002/ece3.11702

**Published:** 2024-07-03

**Authors:** Thomas J. Williams, David Blockley, Andrew B. Cundy, Jasmin A. Godbold, Rebecca M. Howman, Martin Solan

**Affiliations:** ^1^ University of Southampton, National Oceanography Centre Southampton Southampton UK; ^2^ Pinngortitaleriffik Greenland Institute of Natural Resources Nuuk Greenland; ^3^ Québec Océan, Takuvik Joint International Laboratory CNRS Université Laval Quebec City Quebec Canada

**Keywords:** Arctic marine shipping, Arctic trade, bioturbation, fuel contamination, multiple pressures, Northern Sea route

## Abstract

Multiple expressions of climate change, in particular warming‐induced reductions in the type, extent and thickness of sea ice, are opening access and providing new viable development opportunities in high‐latitude regions. Coastal margins are facing these challenges, but the vulnerability of species and ecosystems to the effects of fuel contamination associated with increased maritime traffic is largely unknown. Here, we show that low concentrations of the water‐accommodated fraction of marine fuel oil, representative of a dilute fuel oil spill, can alter functionally important aspects of the behaviour of sediment‐dwelling invertebrates. We find that the response to contamination is species specific, but that the range in response among individuals is modified by increasing fuel concentrations. Our study provides evidence that species responses to novel and/or unprecedented levels of anthropogenic activity associated with the opening up of high‐latitude regions can have substantive ecological effects, even when human impacts are at, or below, commonly accepted safe thresholds. These secondary responses are often overlooked in broad‐scale environmental assessments and marine planning yet, critically, they may act as an early warning signal for impending and more pronounced ecological transitions.

## Graphical abstract

Multiple expressions of climate change are opening access and providing new viable development opportunities in high‐latitude regions, but the vulnerability of species and ecosystems to the effects of fuel contamination associated with increased maritime traffic is largely unknown. Here, we show that low concentrations of the water‐accommodated fraction of marine fuel oil can alter functionally important aspects of the behaviour of sediment‐dwelling invertebrates, even when human impacts are at, or below, commonly accepted safe thresholds.

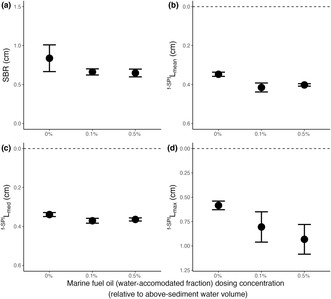

## 1 INTRODUCTION

The Arctic region is experiencing rapid change due to climatic forcing (Burrows et al., 2011; Meredith et al., 2022), resulting in significant transformations in offshore (Horvat et al., 2017), terrestrial (Swanson, 2021) and coastal environments (Ogorodov et al., 2020). The combination of changes in the type, extent and thickness of ice cover (Meier et al., 2014; Pizzolato et al., 2016), retreating glaciers (Vincent et al., 2001), meltwater input (Statham et al., 2008) and water mass dynamics (Fossheim et al., 2015), coupled with warming and ocean acidification (Lam et al., 2016) is modifying short‐ to medium‐term opportunities for the exploitation of abiotic and biotic resources, causing an uplift in transport, trade, tourism and other ship‐based activity across the Arctic region (Bartsch et al., 2021; Dawson et al., 2018; Eguiluz et al., 2016; Pizzolato et al., 2016). In addition, melting and decreasing marine and terrestrial nearshore ice and snow also disrupts traditional transportation routes and methods (e.g. sea ice pathways and sled dogs), resulting in the need for either longer routes or the increased reliance on motorised boats to make long or otherwise inaccessible journeys (Steiro et al., 2020). Such activity brings an increased risk of both acute and chronic marine contamination from deliberate and accidental releases of fuel oils (PAME, 2020) to a seafloor that hosts a diverse (>4600 invertebrate species, Piepenburg et al., 2010) and productive benthic ecosystem (Kędra et al., 2015; Sørensen et al., 2015). Previous infamous oil spills have led to prolonged, catastrophic impacts on marine and coastal systems (Joye, 2015). Even short‐lived, smaller spills can lead to immediate toxicity responses in exposed marine biota and declines in abundance (Brussaard et al., 2016) with likely disruptions in ecosystem productivity if recurrent (Brussaard et al., 2016; Ortmann et al., 2012).

Airborne contaminant bioaccumulation in high‐latitude ecosystems has long been recognised (Alexander, 1995) and, given that the physical characteristics of Arctic marine habitats render them at greater risk of petroleum contamination (Short & Murray, 2011), accidental fuel spills have received considerable scientific attention (Helle et al., 2020). Cold temperatures exert a pronounced influence on hydrocarbon behaviour in seawater, altering compound composition and concentrations by affecting fuel partitioning and diminishing evaporation and degradation rates (Payne et al., 1991; Siron et al., 1993). The presence of ice can also adjust spill dynamics by suppressing wave action, prolonging exposure periods and curbing the spread and dispersion of a spill (Fingas & Hollebone, 2003). As near‐shore habitats remain covered by sea ice for much of the year and can receive runoff from contaminated shoreline sites (Chapman & Riddle, 2003), they are particularly vulnerable to the effects of contamination. Exposure to sublethal concentrations of the water accommodated fraction (WAF) of fuel can affect the behaviour (Brown et al., 2017; Culbertson et al., 2008) and metabolism (Sardi et al., 2017) of invertebrate species, the effects of which can transfer across generations (Lee et al., 2013), while changes in community composition have been observed following exposure to median lethal concentration (LC50) values (Payne et al., 2014).

Despite extensive ecological monitoring programs existing across intertidal, offshore and deep‐sea habitats (Blicher & Arboe, 2021; Sejr et al., 2021; Thyrring et al., 2021), the functional consequences of contaminant‐driven adjustments to species behaviour and alterations in biodiversity for community resilience (Peterson et al., 2003) and ecosystem functioning (Ferrando et al., 2015) are not well constrained. Here, we establish whether exposure to the WAF of marine fuel oil affects the behaviour of three common, but functionally contrasting, sediment‐dwelling invertebrates (the bivalves *Astarte crenata* and *Macoma calcare*a and the polychaete *Cistenides hyperborea*) from the fjordic regions of Greenland. Our a priori expectation was that sediment particle reworking activity—an important mediator of benthic biogeochemical processes and other sediment properties—would broadly reflect interspecific differences in lifestyle traits, but that subtle aspects of reworking behaviour would be modified by the presence of marine fuel oil. Our motivation was to highlight the potential that additional pressures associated with increased human activity may have in high‐latitude regions that are already undergoing substantive transformation.

## 2 MATERIALS AND METHODS

### 2.1 Sediment and invertebrate collection

Sediment [mean ± SE, *n* = 4: D_50_ = 150.25 ± 23.16 μm (D_10_–D_90_, 21.8 ± 2.91–418.5 ± 16.56 μm); organic matter content = 0.30 ± 0.07%] and individuals of the bivalves *Astarte crenata* and *Macoma calcarea*, and the polychaete *Cistenides hyperborea*, were collected from the inner Kobbefjord (64°08.364′ N, 51°23.621′ W; 12 m water depth) using a van Veen grab (0.1 m^2^) deployed from the r.v *Age V Jensen II*. Sediment was sieved (1 mm mesh) in a seawater bath to remove any macrofauna and allowed to settle to retain the fine fraction (<63 μm). Sediment particle size frequency distributions (see Figure S1) were determined optically using a Malvern Mastersizer 3000 LASER diffraction sizer at the School of Geography and Environmental Science, University of Southampton. Briefly, samples were broken down from aggregates using a surface active cleaning agent (Decon™) and a rubber pestle and suspended in distilled water during analysis. Between samples, the instrument was cleaned using pre‐programmed standard settings of three rinses using tap and then distilled water. These data were used to resolve mean particle size, sorting, skewness and kurtosis (Folk, 1974) using GRADISTAT v9.1 (Blott & Pye, 2001; see Table S1). Loss on ignition was used to estimate sediment organic matter content (%; Lamb, 2005). Here, the weight of clean and labelled crucibles was recorded using a Mettler Toledo Analytical Balance (±0.0001 g) prior to the weighing of each wet sediment sample. The crucibles were overnight dried at 105°C using a Gallenkamp Hotbox Oven (Size one), then heated to 550°C for 2 h and to 950°C in a Carbolite Muffle Furnace.

### 2.2 Experimental design and set‐up

We experimentally assess whether three numerically dominant and functionally important benthic invertebrate species (the bivalves *Astarte crenata* and *Macoma calcarea* and the polychaete *Cistenides hyperborea*), from inner Kobbefjord, Greenland, respond to low concentrations of the WAF (or soluble) of marine fuel oil typically generated by wind and/or current turbulent mixing (Lewis et al., 2008). We placed these species in transparent square glass aquaria (internal dimensions, LWH; 11.0 × 11.0 × 23.5 cm) that were each filled with ~11 cm (1.331 L) sieved sediment and overlain with ~9 cm (1.08 L) of seawater (salinity, 33 at 12 m depth; Sørensen et al., 2015) and maintained in a temperature‐controlled water bath (5 ± 1°C, following Solan et al., 2020, see Figure S2) in the dark. We set the abundance and biomass (mean ± SE) of species within each aquarium to levels indicative of those found in inner Kobbefjord: *Astarte crenata*, three ind. and 11.61 ± 0.31 g aquarium^−1^ (= 248 ind./959 g m^−2^); *Macoma calcarea*, four ind. and 7.51 ± 0.48 g aquarium^−1^ (= 331 ind./621 g m^−2^) and *Cistenides hyperborea*, six ind. and 3.09 ± 0.14 g aquarium^−1^ (= 496 ind./255 g m^−2^); see Figure S3. Our experimental design required a total of 36 aquaria (3 species × 3 marine fuel oil concentrations × 4 replicates). Each aquarium was continually aerated and maintained for 7 days.

### 2.3 Introduction of marine fuel

Marine fuel oil was sourced directly from a local marine supplier in Nuuk, Greenland. A low‐energy water‐accommodated fraction (LE‐WAF) was prepared following established guidelines from the Chemical Response to Oil Spills: Ecological Research Forum (CROSERF), using a modified method from that previously described, in closed vessels (Faksness & Altin, 2017). Specifically, an oil‐to‐water ratio of 1:40 was used, with a reduced contact time of 2 h (due to laboratory and experimental constraints). At this contact ratio, the system is assumed to be ‘saturated’ and therefore represents a conservative estimate of concentrations introduced by vessel traffic (Faksness & Altin, 2017). Using LE‐WAF is preferential to introducing fuel oil directly as it avoids the generation of oil droplets, which may lead to dosing variability between treatments and enhanced exposure through organism adherence (Hansen et al., 2021). Following preparation, the WAF of the oil was removed and used for dose treatments. Each species was maintained in one of the three marine fuel oil concentrations (*n* = 4 replicates species^−1^ fuel concentration^−1^): 0% (control), 0.1% and 0.5% marine fuel oil (i.e. 1 mL and 5 mL L^−1^ seawater; WAF, Table 1). These concentrations are at the lower end of dosing rates observed to generate sub‐lethal, developmental or reproductive effects in a range of marine organisms, including polychaetes (e.g. Lewis et al., 2008; Pereira et al., 2018), and are designed to simulate organism exposure to the WAF (or soluble) generated by turbulent mixing by winds and currents (Lewis et al., 2008). A water sample was taken from the WAF immediately before dosing to determine WAF composition and hydrocarbon concentration. The sample was sealed using parafilm, stored in darkness during transport and analysed for total petroleum hydrocarbons (aliphatic and aromatic fractions) via gas chromatography of solvent extracts (FID) and total and individual PAHs via gas chromatography linked mass spectrometry (GC–MS) at an accredited commercial laboratory.

**TABLE 1:** Summary of hydrocarbon dosing concentrations.

Component	Concentration in undiluted LE‐WAF (mg L^−1^)	Concentration in 0.1% dosing experiments (mg L^−1^)	Concentration in 0.5% dosing experiments (mg L^−1^)
Total PAHs	<0.1	<0.1	<0.1
Total TPH (C10–C40)	21.1	0.021	0.106
TPH Aliphatic (C8–C10)	1.43	0.001	0.007
TPH Aliphatic (C10–C12)	2.84	0.003	0.014
TPH Aliphatic (C12–C16)	0.10	0.0001	0.001
TPH Aliphatic (C16–C21)	3.01	0.003	0.015
TPH Aliphatic (C21–C40)	3.79	0.004	0.019
TPH Aromatic (C8–C10)	0.35	0.0004	0.002
TPH Aromatic (C10–C12)	<0.1	<0.0001	<0.001
TPH Aromatic (C12‐C16)	5.64	0.006	0.028
TPH Aromatic (C16–C21)	<0.1	<0.0001	<0.001
TPH Aromatic (C21–C40)	5.74	0.006	0.029

*Note*: Concentrations reported as ‘less than’ are below method detection limits. Individual PAHs (Acenaphthylene, Acenaphthene, Anthracene, Benzo(a)pyrene, Benzo(g,h,i)perylene, Benz(a)anthracene, Benzo(k)fluoranthene, Benzo(b)fluoranthene, Chrysene, Dibenzo(a, h)anthracene, Fluorene, Fluoranthene, Indeno(1,2,3‐c,d)pyrene, Napthalene, Phenanthrene and Pyrene) were also analysed and were below detection limits (0.01 mg L^−1^) in all fractions. TPH = total petroleum hydrocarbons, including split by aliphatic and aromatic fractions and carbon chain length.

Abbreviation: LE‐WAF, Low energy Water‐Accommodated Fraction.

### 2.4 Quantification of bioturbation behaviour

Faunal reworking of sediment particles (bioturbation) was estimated using non‐invasive sediment profile imaging (f‐SPI) (Solan et al., 2004) of the redistribution of a fluorescent dyed particle tracer, imaged under ultraviolet (UV) light [50 g aquarium^−1^, green colour; particle size, D_50_ = 301 μm (D_10_–D_90_, 206–438 μm); Glass Pebbles Ltd, UK] after 7 days. Images (94 μm pixel^−1^ resolution) were taken of all four aquarium sides using a digital camera (CANON 400D) in a UV‐illuminated dark box and stitched together in Adobe Photoshop CS6 (version 13.0 x64). The vertical distribution of luminophores was determined from the stitched images (see Figures S4 and S5) using a custom‐made, semi‐automated macro that runs within ImageJ (v. 1.47), a Java‐based public domain program (Schneider et al., 2012). From these data, the mean (^f‐SPI^L_mean_, time‐dependent indication of mixing), median (^f‐SPI^L_med_, typical short‐term depth of mixing), and maximum (^f‐SPI^L_max_, maximum extent of mixing over the long‐term) mixed depth of particle redistribution were calculated. In addition, the maximum vertical deviation of the sediment–water interface (surface boundary roughness, SBR) provided an indication of surficial activity (Hale et al., 2014).

### 2.5 Statistical analysis

Analysis of variance (ANOVA) models were developed for each dependent variable (SBR, ^f‐SPI^L_mean,_^f‐SPI^L_median,_^f‐SPI^L_max_) to determine the effects of the marine fuel oil concentration (three levels: 0%, 0.1% and 0.5%, WAF; replication, *n* = 4 species^−1^). As our focus was to determine species‐specific responses to marine fuel oil at indicative natural densities, we used an independent model for each species to avoid the confounding effects caused by differences in species abundance and biomass. Model assumptions were visually assessed using standardised residuals vs fitted value plots, Q–Q plots and Cook's distance (Zuur et al., 2010). Where there was a violation of homogeneity of variance, we used a *varIdent* variance–covariance structure and generalised least squares (GLS) estimation (Pinheiro & Bates, 2000; West et al., 2014) to allow residual spread to vary among groups. We determined the optimal fixed effects structure using backward selection informed by Akaike information criteria (AIC) and inspection of model residual patterns. For the GLS analysis, we determined the optimal variance–covariance structure using restricted maximum‐likelihood (REML) estimation by comparing the initial ANOVA model without variance structure to equivalent GLS models incorporating specific variance terms. These models were compared against the initial ANOVA model using AIC informed by visualisation of model residuals. We determined the optimal fixed structure of the most suitable model by applying backward selection using the likelihood ratio test with maximum‐likelihood (ML) estimation (West et al., 2014; Zuur et al., 2010). Details of initial and minimal adequate models (Models S1–S12), as well as model coefficient tables, are provided in the Supporting Information.

## 3 RESULTS

The activities of all three species used in our study resulted in the vertical mixing of luminophore tracers, with subtle differences in the form of the profile (Figure S5) reflecting differences in species behaviour. Surface boundary roughness ranged from 0.48 cm (0% marine fuel oil) to 1.21 cm (0% marine fuel oil) in *Astarte crenata*, from 0.48 cm (0% marine fuel oil) to 1.24 cm (0.5% marine fuel oil) in *Macoma calcarea* and from 0.39 cm (0.1% marine fuel oil) to 1.03 cm (0% marine fuel oil) in *Cistenides hyperborea*. Similarly, the range of the mean [^f‐SPI^L_mean_: *A*. *crenata*, from 0.32 cm (0% marine fuel oil) to 0.47 cm (0.1% marine fuel oil); *M*. *calcarea*, from 0.35 cm (0.1% marine fuel oil) to 0.68 cm (0% marine fuel oil) and *C*. *hyperborea*, from 0.35 cm (0.1% marine fuel oil) to 0.51 cm (0.5% marine fuel oil)], median [^f‐SPI^L_med_: *A*. *crenata*, from 0.31 cm (0% marine fuel oil) to 0.40 cm (0.1% marine fuel oil); *M*. *calcarea*, from 0.28 cm (0.5% marine fuel oil) to 0.44 cm (0.5% marine fuel oil) and *C*. *hyperborea*, from 0.33 cm (0.1% marine fuel oil) to 0.43 cm (0.5% marine fuel oil)] and maximum [^f‐SPI^L_max_: *A*. *crenata*, from 0.50 cm (0% marine fuel oil) to 1.33 cm (0.5% marine fuel oil); *M*. *calcarea*, from 0.66 cm (0.1% marine fuel oil) to 3.90 cm (0% marine fuel oil) and *C*. *hyperborea*, from 0.57 cm (0.5% marine fuel oil) to 1.13 cm (0.1% marine fuel oil)] depths of mixing varied across marine fuel oil concentrations.

Analysis of total biomass across marine fuel oil concentration treatments confirmed no differences in biomass for each species (*A*. *crenata*: *F*_2,9_ = 0.271, *p* = .765; *M*. *calcarea*, *F*_2,9_ = 0.850, *p* = .463; *C*. *hyperborea*, *F*_2,9_ = 0.860, *p* = .455), negating the need to include biomass as a random effect in our statistical models.

**FIGURE 1:**
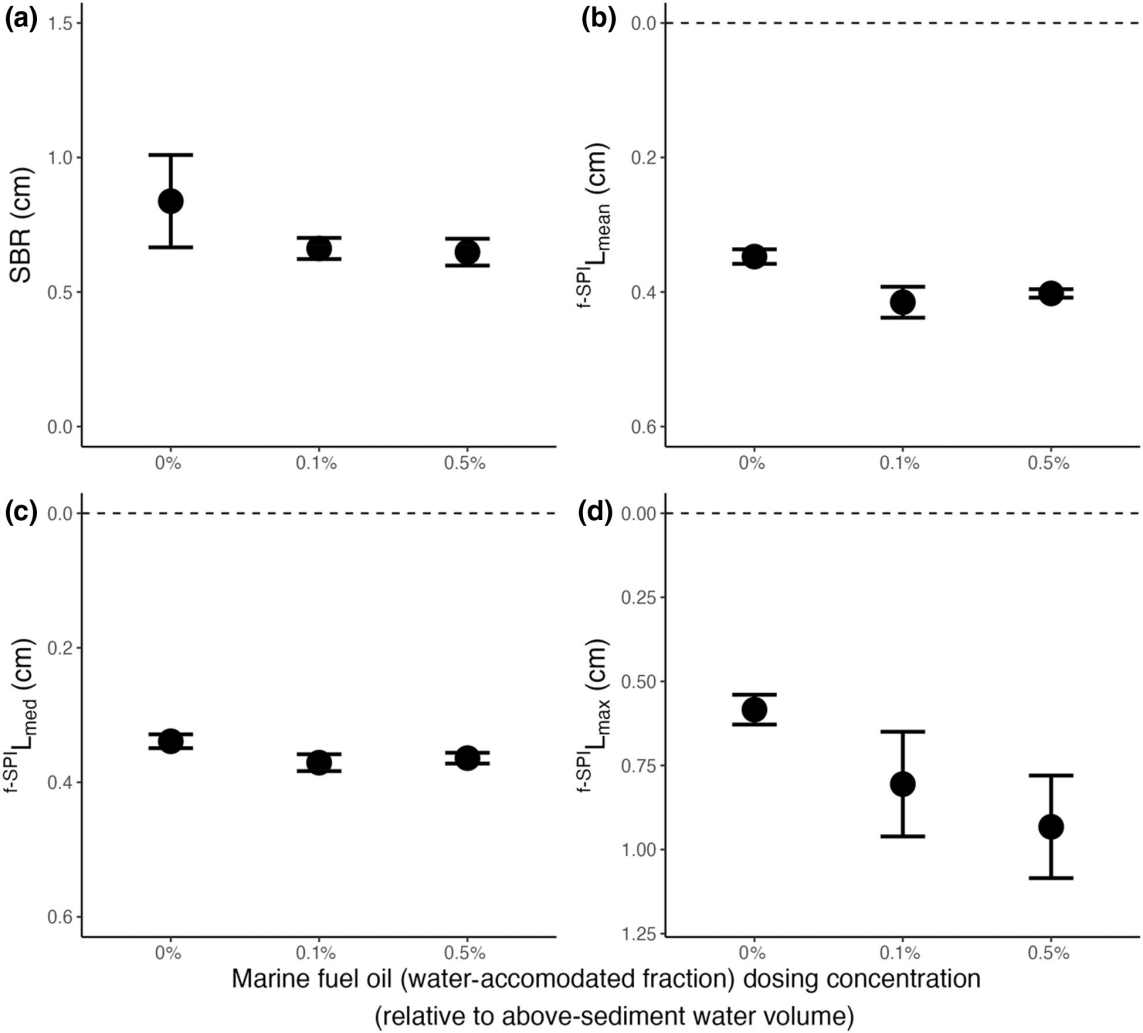
Summary of the bioturbation behaviour of the bivalve, *Astarte crenata*, in the presence of different concentrations of the water‐accommodated fraction of marine fuel oil on (mean ± SE) (a) surface boundary roughness (cm) and the depth of (b) ^f‐SPI^L_mean_ (cm), (c) ^f‐SPI^L_med_ (cm), (d) ^f‐SPI^L_max_ (cm). In panels (b–d), horizontal dashed lines represent the position of the sediment–water interface. Sediment profile images and associated luminophore distribution profiles are presented in Supporting Information, Figure S5. LE‐WAF concentrations for different fuel oil hydrocarbon components are shown in Table 1.

In aquaria containing *A*. *crenatta*, SBR and median mixing depth (^f‐SPI^L_med_) were unaffected by marine fuel oil (intercept‐only models: SBR, L ratio = 1.300, *df* = 2, *p* = .523; ^f‐SPI^L_med_, L ratio = 4.636, *df* = 2, *p* = .096), while mean mixing depth became notably shallower (^f‐SPI^L_mean_, L ratio = 9.430, *df* = 2, *p* < .01) and maximum mixing depth, although marginally, showed evidence of a deepening (^f‐SPI^L_max_, L ratio = 5.770, *df* = 2, *p* = .056; Figure 1). Closer examination of model coefficients revealed a difference in ^f‐SPI^L_mean_ between marine fuel oil concentrations of 0% and 0.1% (coefficient ± SE: 0.068 ± 0.025, *t* = 2.670, *p* < .05) and 0% and 0.5% (coefficient ± SE: 0.055 ± 0.012, *t* = 4.396, *p* < .01) but not between 0.1% and 0.5% (coefficient ± SE: −0.013 ± 0.024, *t* = 0.548, *p* = .597). Specifically, ^f‐SPI^L_mean_ increased from (mean ± SE) 0.35 ± 0.01 cm in the absence of marine fuel oil to 0.42 ± 0.02 and 0.40 ± 0.01 cm at concentrations of 0.1% and 0.5%, respectively. The maximum mixing depth (mean ± SE) increased from 0.58 ± 0.04 cm in the absence of marine fuel oil to 0.81 ± 0.16 cm under 0.1% and 0.93 ± 0.16 cm under 0.5% of marine fuel oil. Model coefficients for ^f‐SPI^L_max_ demonstrated no difference between marine fuel oil concentrations of 0% and 0.1% (coefficient ± SE: 0.221 ± 0.162, *t* = 1.368, *p* = .205) or between 0.1% and 0.5% (coefficient ± SE: 0.127 ± 0.218, *t* = 0.583, *p* = .574), but there was some weak evidence for a deepening of ^f‐SPI^L_max_ between 0% and 0.5% (coefficient ± SE: 0.349 ± 0.159, *t* = 2.195, *p* = .056).

**FIGURE 2:**
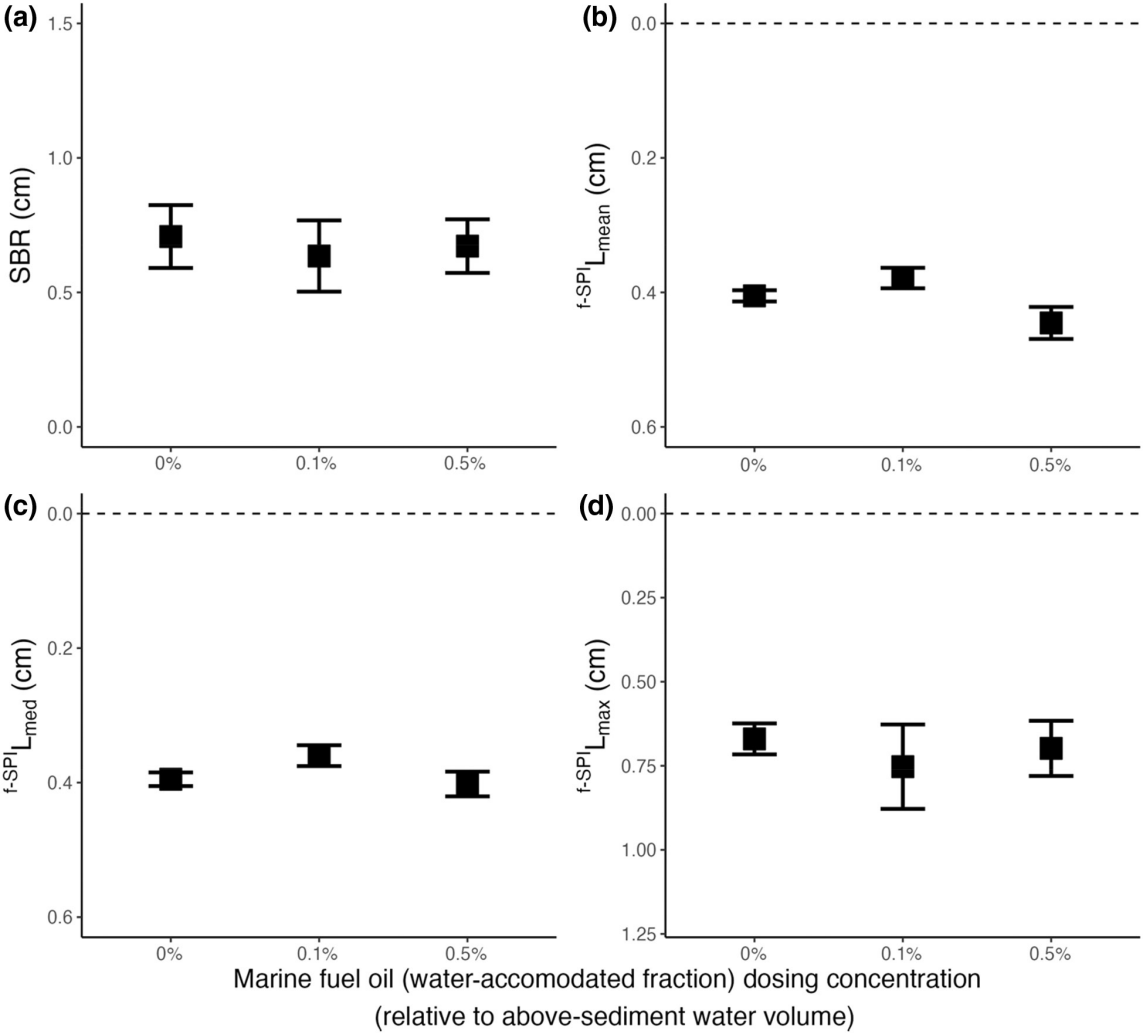
Summary of the bioturbation behaviour of the polychaete, *Cistenides hyperborea*, in the presence of different concentrations of the water‐accommodated fraction of marine fuel oil on (mean ± SE) (a) surface boundary roughness (cm) and the depth of (b) ^f‐SPI^L_mean_ (cm), (c) ^f‐SPI^L_med_ (cm), (d) ^f‐SPI^L_max_ (cm). In panels (b–d), horizontal dashed lines represent the position of the sediment–water interface. Sediment profile images and associated luminophore distribution profiles are presented in Supporting Information, Figure S5. LE‐WAF concentrations for different fuel oil hydrocarbon components are shown in Table 1.

For aquaria containing *C*. *hyperborea*, SBR, median mixing depth and maximum mixing depth were independent of marine fuel oil (intercept‐only models: SBR, L ratio = 0.222, *df* = 2, *p* = .895; ^f‐SPI^L_median_, L ratio = 4.039, *df* = 2, *p* = .133; ^f‐SPI^L_max_, L ratio = 0.540, *df* = 2, *p* = .763). However, there was some weak evidence that the mean mixing depth extends deeper in the presence of marine fuel oil (^f‐SPI^L_mean_, L ratio = 5.433, *df* = 2, *p* = .067; Figure 2). This observation is driven by a deepening of ^f‐SPI^L_mean_ between 0.1% and 0.5% of marine fuel oil (coefficient ± SE, 0.067 ± 0.029, *t* = 2.364, *p* < .05), but we found no difference in ^f‐SPI^L_mean_ between 0% and 0.1% (coefficient ± SE, −0.026 ± 0.017, *t* = 1.524, *p* = .162) or 0% and 0.5% (coefficient ± SE, 0.040 ± 0.025, *t* = 1.599, *p* = .144) of marine fuel oil. Specifically, ^f‐SPI^L_mean_ was 0.41 ± 0.01 cm in the absence of marine fuel oil, 0.38 ± 0.02 cm under 0.1% and 0.45 ± 0.02 cm under 0.5% marine fuel oil.

**FIGURE 3:**
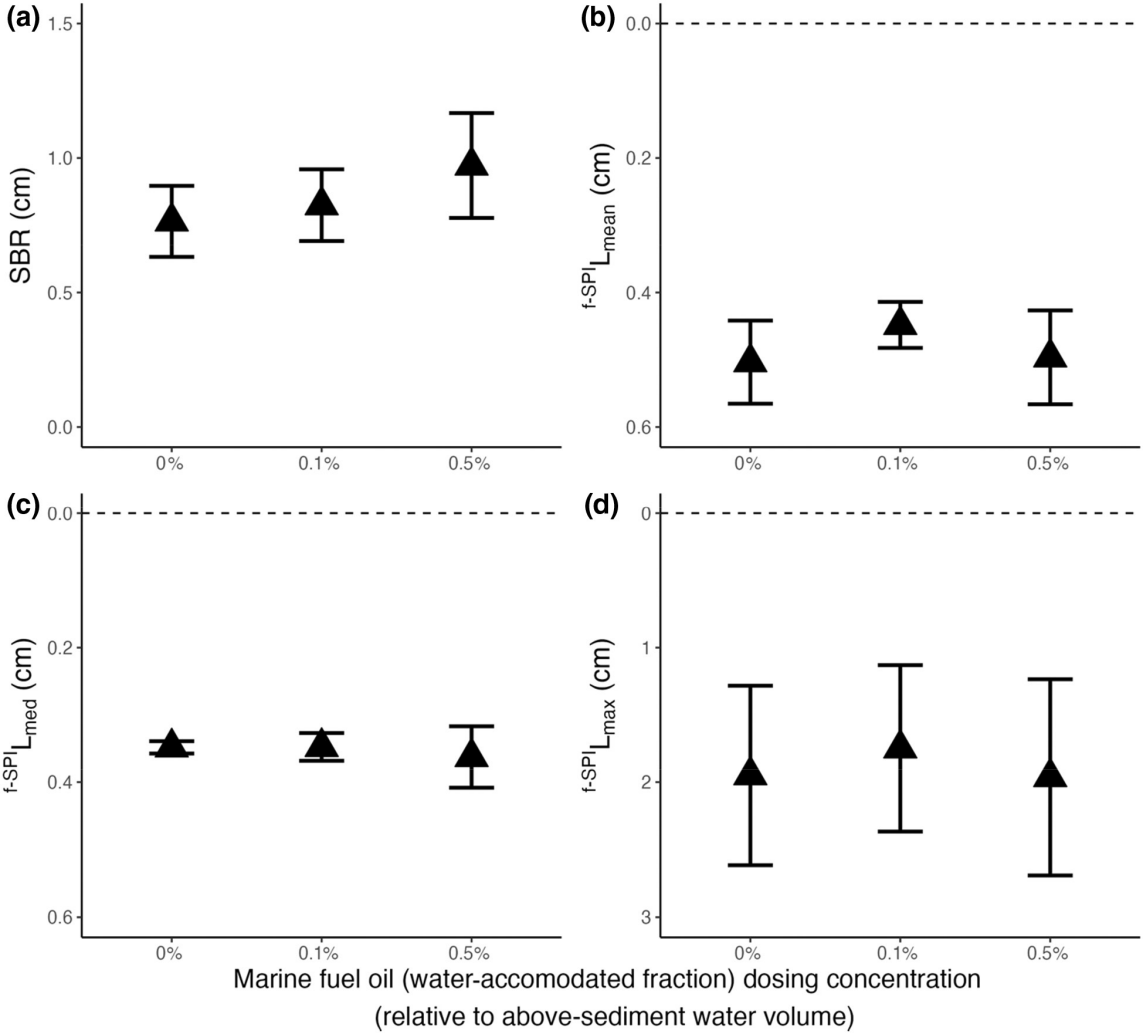
Summary of the bioturbation behaviour of the bivalve, *Macoma calcarea*, in the presence of different concentrations of the water‐accommodated fraction of marine fuel oil on (mean ± SE) (a) surface boundary roughness (cm) and the depth of (b) ^f‐SPI^L_mean_ (cm), (c) ^f‐SPI^L_med_ (cm), (d) ^f‐SPI^L_max_ (cm). In panels (b–d), horizontal dashed lines represent the position of the sediment–water interface. Sediment profile images and associated luminophore distribution profiles are presented in Supporting Information, Figure S5. LE‐WAF concentrations for different fuel oil hydrocarbon components are shown in Table 1.

In contrast to *A*. *crenata* and *C*. *hyperborea*, we found no evidence that the bioturbation behaviour of *M*. *calcarea* is affected by the applied concentrations of marine fuel oil (intercept‐only models: SBR, L ratio = 1.022, *df* = 2, *p* = .600; ^f‐SPI^L_mean_, L ratio = 1.082, *df* = 2, *p* = .582; ^f‐SPI^L_median_, L ratio = 0.142, *df* = 2, *p* = .931; ^f‐SPI^L_max_, L ratio = 0.093, *df* = 2, *p* = .955; Figure 3). However, absolute values of SBR and median mixing depth (mean ± SE) did show a directional trend, increasing from 0.77 ± 0.13 cm and 0.35 ± 0.01 cm in the absence of marine fuel oil to 0.97 ± 0.20 cm and 0.36 ± 0.05 cm under 0.5% marine fuel oil, respectively.

## 4 DISCUSSION

Our findings demonstrate that the accumulation of the WAF of marine fuel oil can lead to shifts in functionally important aspects of sediment‐dwelling invertebrate behaviour, although these effects are species‐specific and are not necessarily linear as contamination concentrations increase. Such changes in behaviour—here burying deeper, indicative of classic avoidance behaviour (Maire et al., 2010)—are known to influence the fate of sediment‐associated pollutants (Tian et al., 2019; Tong et al., 2019) and can be sufficient to change the functional role of the species (Wohlgemuth et al., 2017). Burying deeper to avoid contamination in the surficial layers can enhance particle mixing but can have negative consequences for the growth and survival of individuals over an extended period of time (de Goeij & Luttikhuizen, 1998). Conversely, altered surficial reworking activity, as observed here, can either confine sediment‐associated contaminants to the sediment surface or stimulate both downward and outward contaminant transfer (Gilbert et al., 1994), while the presence of the contaminant can directly disrupt important microbial‐mediated pathways (e.g. biogeochemical cycling, Gilbert et al., 1997).

An important aspect of our findings is that not all species changed behaviour in response to the presence of marine fuel oil, perhaps reflecting differences in vulnerability or thresholds. However, while these results are consistent with the findings elsewhere on contamination (Dorgan et al., 2020; O'Brien & Keough, 2014), we obtained contrasting responses for two similarly sized bivalves that ordinarily adopt very similar functional roles. Though identifying the mechanistic basis for this difference is beyond the scope of the present study, having species that respond differently to the same pressure could be viewed as being ecologically advantageous, as it increases the capacity of a system to recover following chronic disturbance events (Duffy, 2009), especially amongst organisms that coexist (Pages‐Escola et al., 2018) or, as in this case, perform overlapping functional roles. Moreover, communities with diverse response capacities have higher probabilities of including organisms that persist under specific environmental conditions and functionally compensate following species loss (Bernhardt & Leslie, 2013; Thomsen et al., 2017, 2019), minimising the impact on linked ecosystem services when environmental conditions fluctuate over time (Truchy et al., 2015). Although the effects of the conservative contamination event simulated here were relatively subtle, we did note a change in intra‐specific variation of sediment reworking activity akin to other studies on responses to pressures in high‐latitude marine invertebrates (Williams et al., 2024). Intra‐specific variation in trait expression plays a pivotal role in population maintenance (Bolnick et al., 2011; Dingemanse & Wolf, 2013), adaptation to dynamic environmental conditions (Henn et al., 2018; Sanders et al., 2024) and contributes to the stability of ecosystem functioning (Wright et al., 2016). Consequently, alterations in this variability bear significant implications for broader ecological processes, potentially influencing species interactions, community dynamics and overall ecosystem resilience (Des Roches et al., 2018; McEntire et al., 2022).

Our study provides evidence that species responses to novel and/or unprecedented levels of anthropogenic activity associated with the opening up of high‐latitude regions can have substantive ecological effects, even when the source of perturbation is below acute toxicity thresholds. Investment in the Arctic is expected to be in the order of billions of dollars over the next decade (Kudryashova et al., 2019) as nations not only take advantage of economic opportunities presented by the rapid opening up of the region (O'Garra, 2017) but also prepare for the challenges associated with its governance (Ebinger & Zambetakis, 2009). In Greenland, where this study is grounded, opportunistic resource exploration following the convergence of climate warming and glacial retreat has strong indigenous backing (Bendixen et al., 2022) but concerns persist regarding the sustainability of such decisions (Hanaček et al., 2022) and the possible negative feedback of expanded infrastructure on the climate (Masson‐Delmotte et al., 2012) and local ecosystems (Bendixen et al., 2019), the latter of which is already affected by regional climate change (Gross, 2018). Though we find mixed results in the responses of benthic biodiversity to low marine fuel oil contamination, it is important to note that the responses observed here are at contaminant concentrations below the threshold of current environmental risk assessments (Faksness & Altin, 2017) and at the lower end of previously observed effects (Pereira et al., 2018). Hence, these observations may act as an early warning for impending and more pronounced ecological transitions. As contaminants accumulate, either directly from spillages associated with shipping activity or resuspension via dredging (Hedge et al., 2009), it follows that there will likely be more pronounced species responses, including demographic or functional transitions where lethal concentrations are reached (O'Brien & Keough, 2014), or more complex ecological outcomes when contaminants co‐occur and lead to non‐additive responses (Millward et al., 2004). Identifying general response patterns to sub‐lethal contamination in the Arctic environment is critical for reliable assessments of ecosystem health (Eldridge et al., 2022) and demands effective management of the expansion of human activity within the context of biodiversity and climate change within the region (Wu et al., 2017).

## AUTHOR CONTRIBUTIONS

**Thomas J. Williams:** Conceptualization (lead); formal analysis (equal); funding acquisition (lead); investigation (equal); methodology (equal); writing – original draft (lead); writing – review and editing (equal). **David Blockley:** Conceptualization (equal); funding acquisition (equal); project administration (equal); writing – review and editing (equal). **Andrew B. Cundy:** Conceptualization (equal); funding acquisition (equal); investigation (equal); methodology (equal); writing – review and editing (equal). **Jasmin A. Godbold:** Conceptualization (equal); formal analysis (equal); funding acquisition (equal); investigation (equal); methodology (equal); writing – original draft (equal); writing – review and editing (equal). **Rebecca M. Howman:** Formal analysis (equal); funding acquisition (equal); investigation (equal); methodology (equal); writing – review and editing (equal). **Martin Solan:** Conceptualization (lead); data curation (equal); formal analysis (equal); funding acquisition (lead); investigation (equal); methodology (equal); project administration (lead); writing – original draft (lead); writing – review and editing (equal).

## CONFLICT OF INTEREST STATEMENT

The authors declare that there are no competing interests.

### OPEN RESEARCH BADGES

This article has earned an Open Data badge for making publicly available the digitally‐shareable data necessary to reproduce the reported results. The data is available at https://doi.org/10.7910/DVN/VECWVX.

## Supporting information


Appendix S1.

## Data Availability

All f‐SPI images and their corresponding extracted luminophore profiles are available from Harvard Dataverse at https://doi.org/10.7910/DVN/VECWVX. This archive also includes the derived data used for statistical analysis, which are also provided in the Supporting Information, Table S2.
