# Supplementary material for: Dilute concentrations of maritime fuel can modify sediment reworking activity of high‐latitude marine invertebrates
Source: Ecol Evol. 2024 Jul 3;14(7):e11702. doi: 10.1002/ece3.11702 (PMC11222169; doi:10.1002/ece3.11702)
Supplement: Supplementary file 1 — Appendix S1. [file ECE3-14-e11702-s001.docx]

**Appendix**

**Dilute concentrations of maritime fuel can modify sediment reworking activity of high-latitude marine invertebrates**

Thomas J. Williams^1^, David Blockley^2^, Andrew B. Cundy^1^, Jasmin A. Godbold^1^, Rebecca M. Howman^1,3^, Martin Solan^1^

^1^University of Southampton, National Oceanography Centre Southampton, Waterfront Campus, European Way, Southampton, SO14 3ZH, UK

^2^Pinngortitaleriffik, Greenland Institute of Natural Resources, Kivioq 2, 3900 Nuuk, Greenland

^3^Québec Océan, Takuvik Joint International Laboratory CNRS, Université Laval, Quebec City, QC, Canada

**Figure S1 |** Cumulative sediment particle size distributions (n = 4) for sieved (1mm mesh size) sediment collected from the inner Kobbefjord, near Nuuk, Greenland (12m depth, 64°08.364 N, 51°23.621’ W).


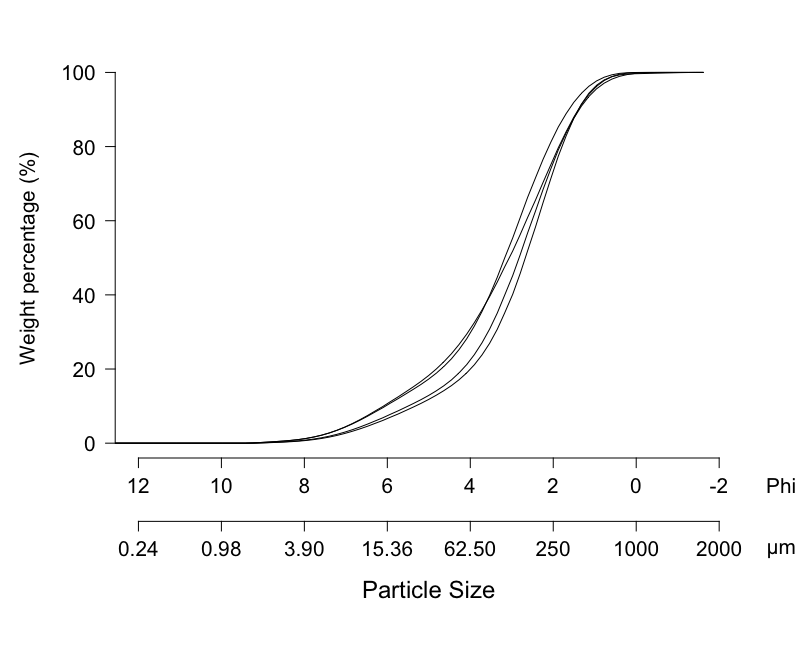


**Figure S2 |** The temperature-controlled insulated water bath housing randomly positioned square glass aquaria prior to faunal addition. Temperature (± 1˚ C) is maintained via continuous circulation of seawater through an external heater-chiller unit. Individual aquaria are supplied with continuous aeration via the rows of taps along the perimeter of the water bath.

**
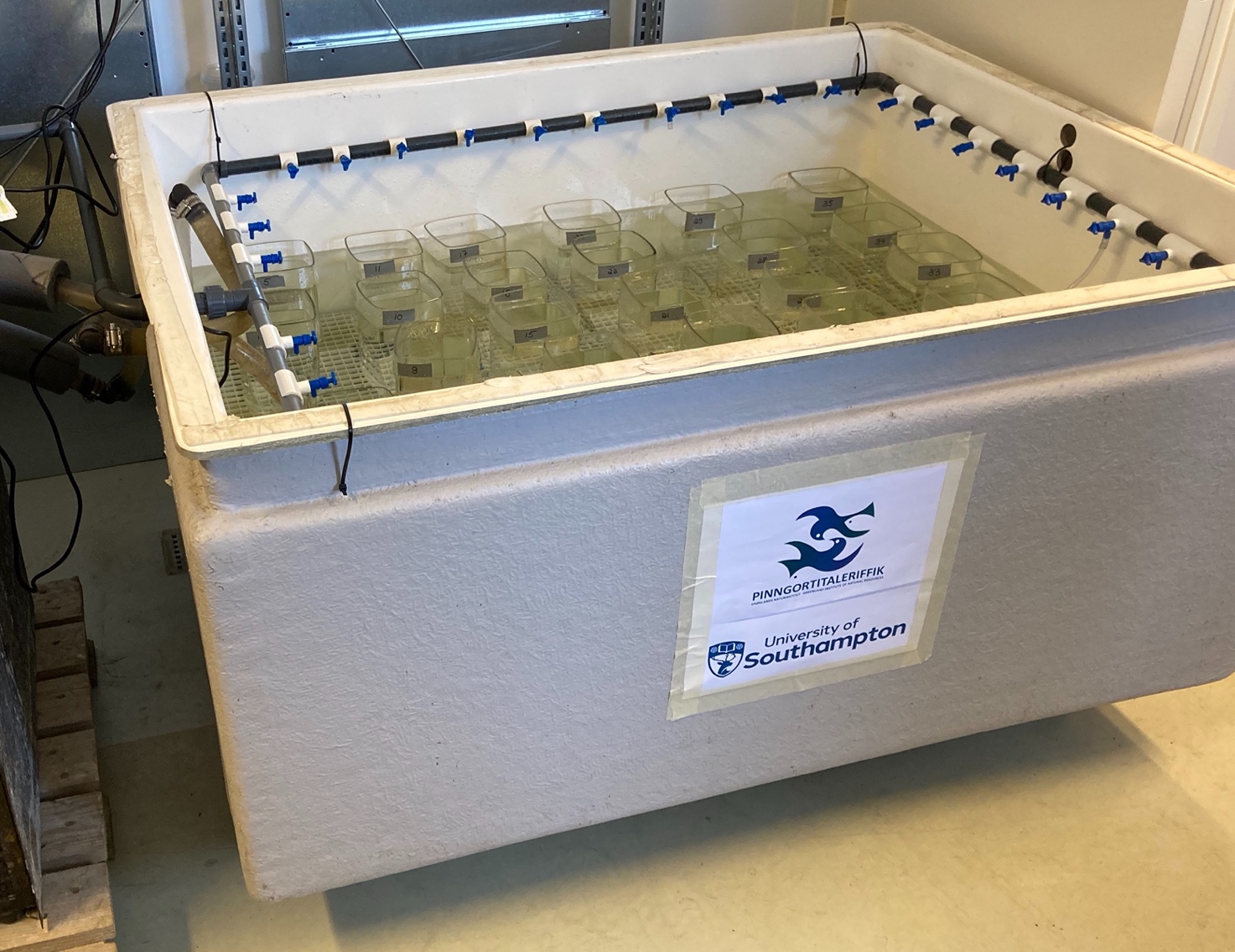
**

**Figure S3** **|** Total biomass aquaria^-1^ for *Astarte crenata* (circle), *Cistenides hyperborea* (square) and *Macoma calcarea* (triangle) for each marine fuel oil (water accommodated fraction) dosing concentration (relative to above-sediment water volume).


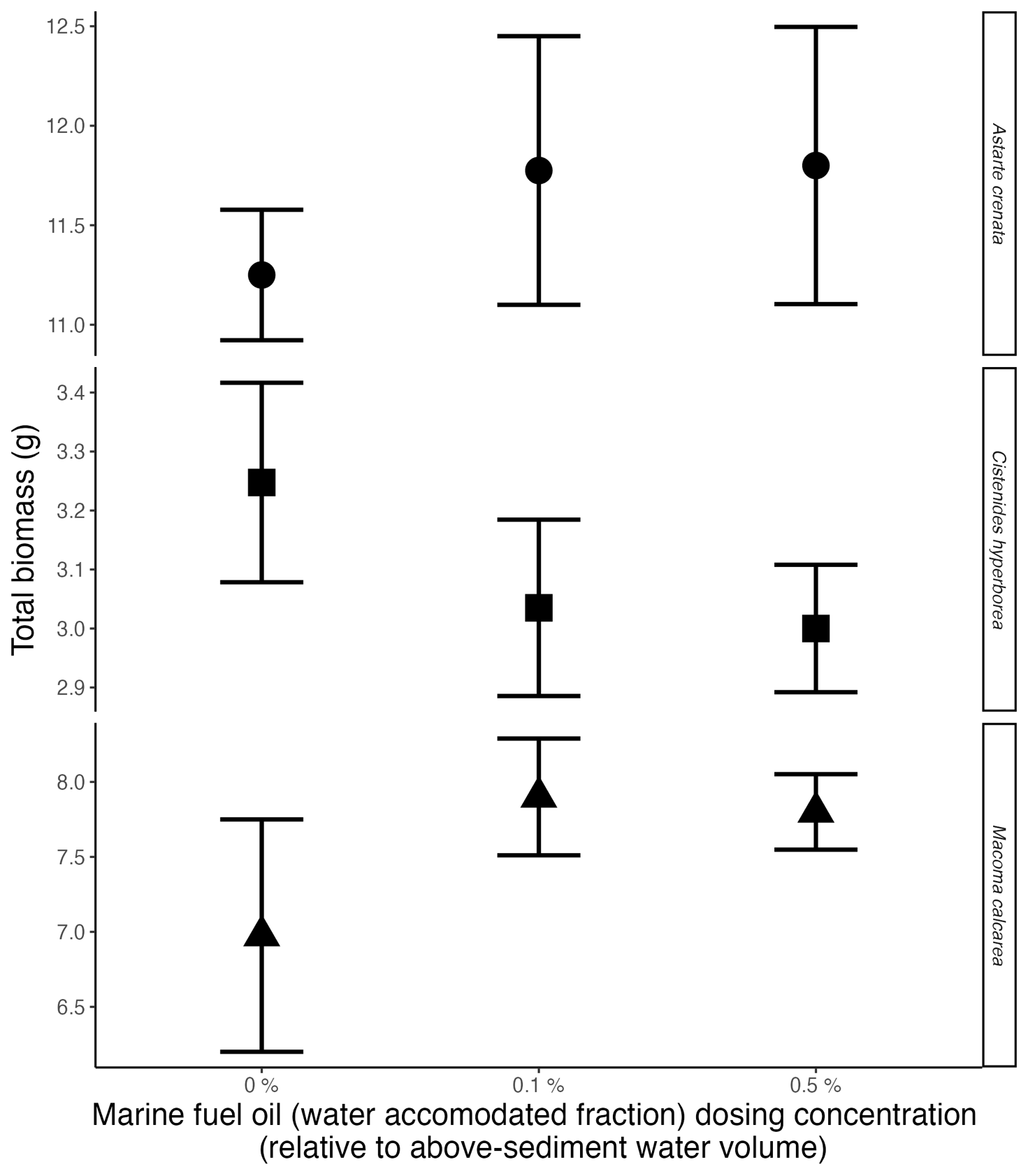


**Figure S4 |** Replicate (n = 4, with one exception of n = 3) f-SPI images for aquaria containing (a-c) *Astarte crenata* exposed to (a) 0%, (b) 0.1% and (c) 0.5% marine fuel oil (water accommodated fraction) dosing concentration (relative to above-sediment water volume). (d-f) *Cistenides hyperborea* exposed to (a) 0%, (b) 0.1% and (c) 0.5 % marine fuel oil concentrations, and (g-i) *Macoma calcarea* exposed to (g) 0%, (h) 0.1% and (i) 0.5% marine fuel oil concentrations. Each image shows the four aquarium sides, each ~10 cm width, stitched together after 7 days. The green coloration is the luminophore tracer.

Figure S4(a)


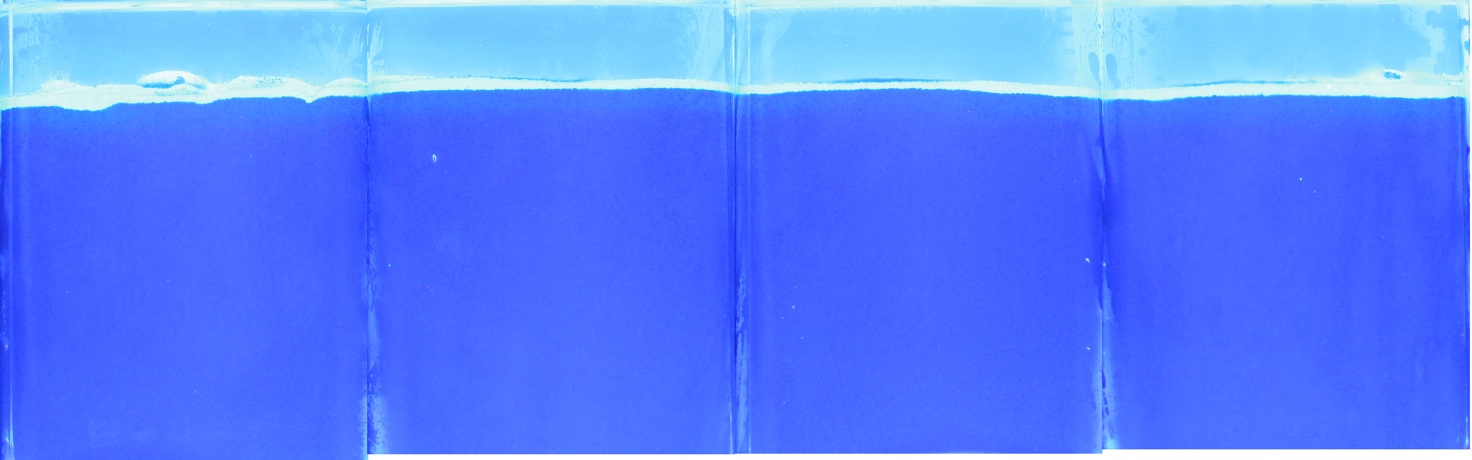


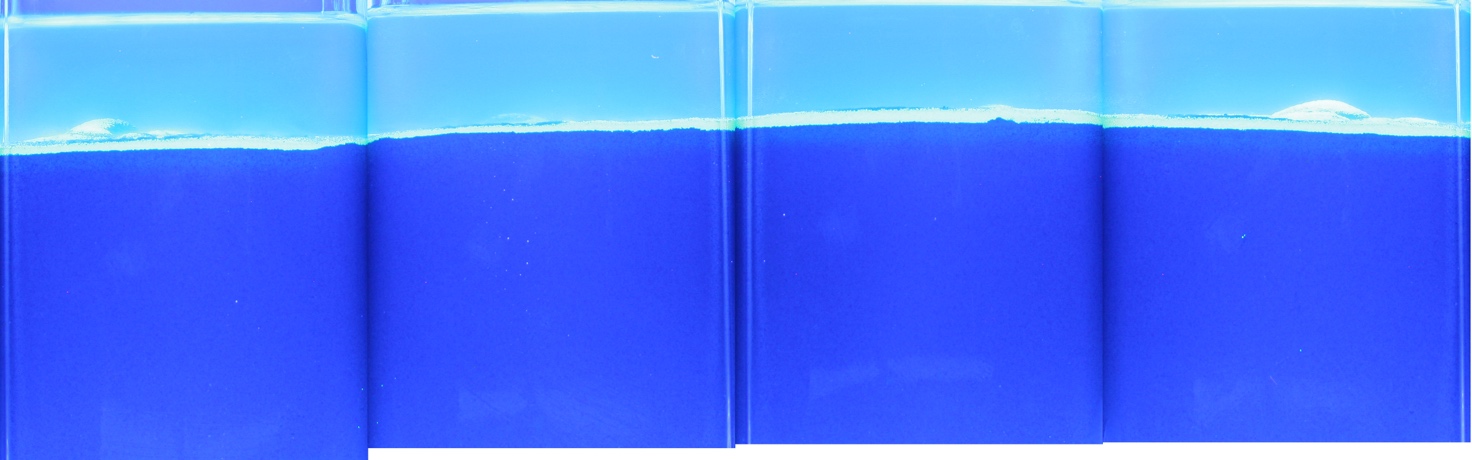


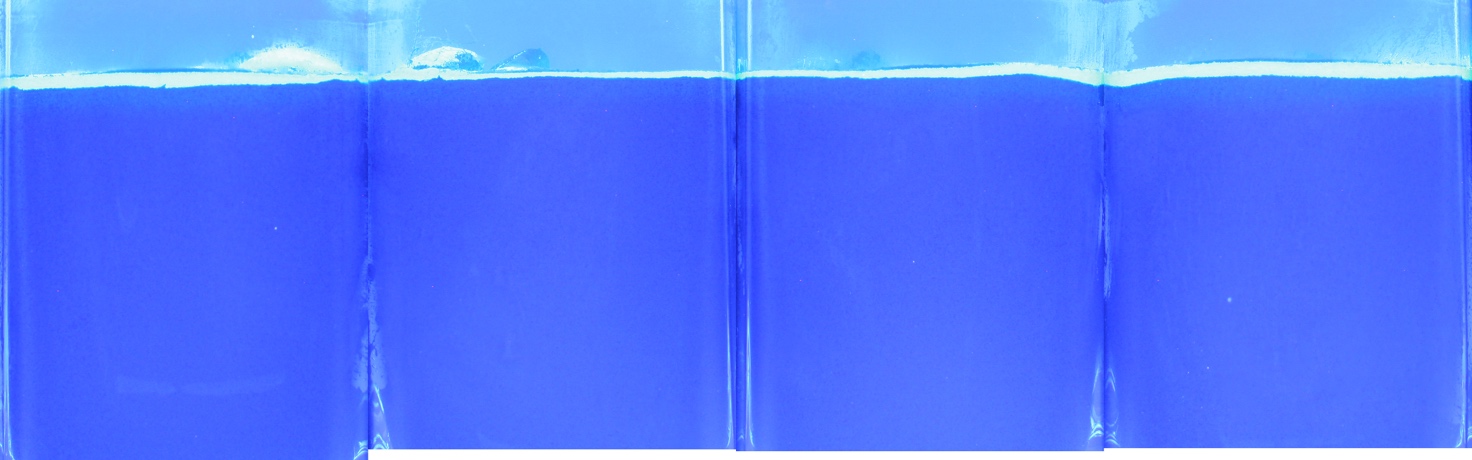


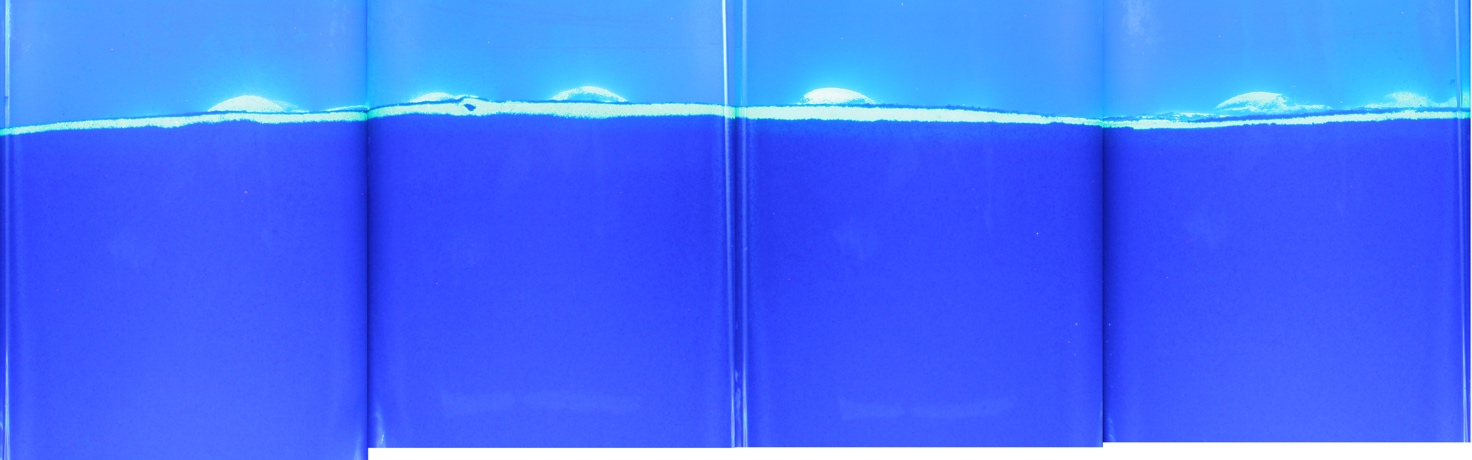


Figure S4(b)


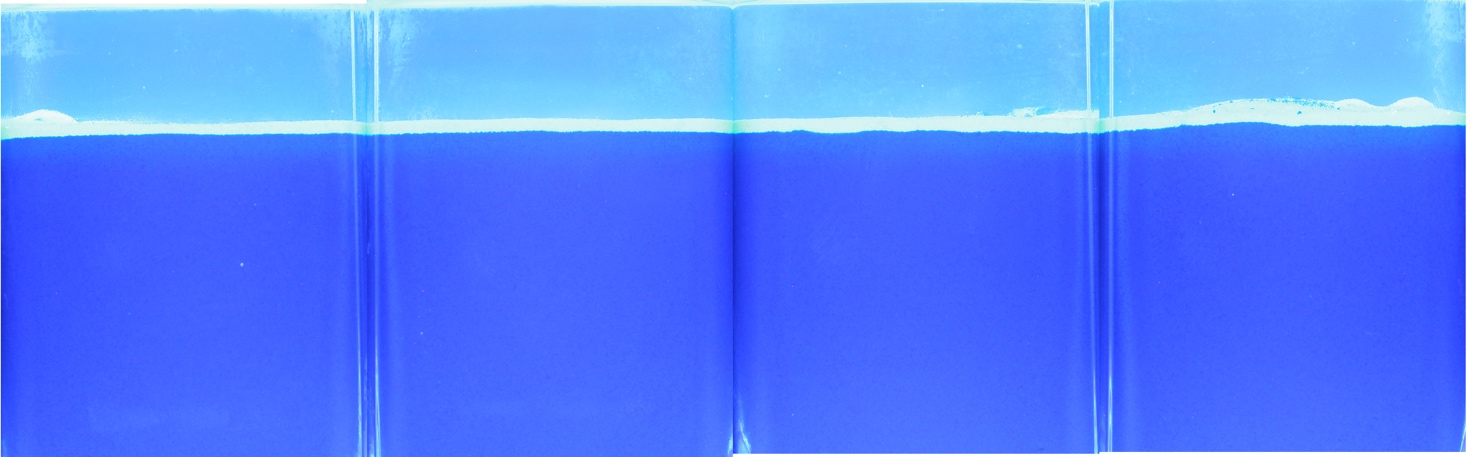


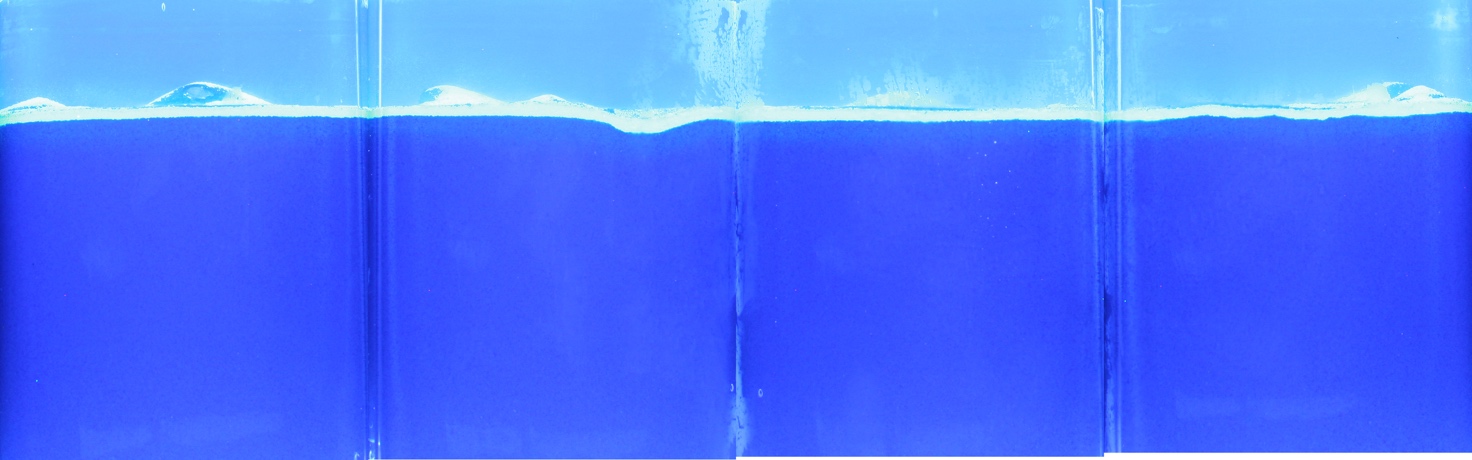


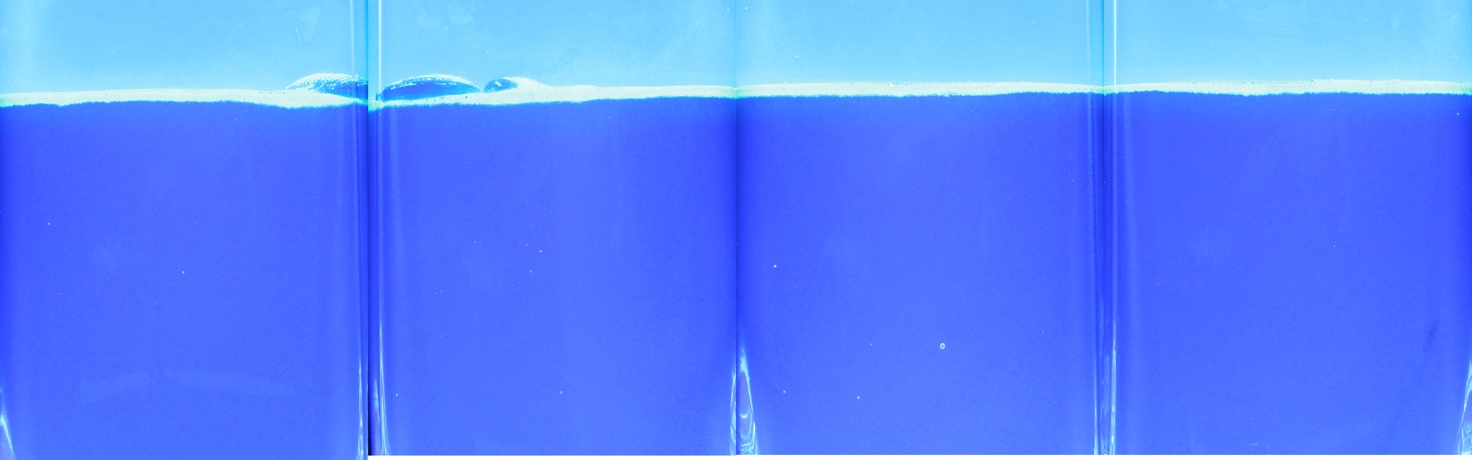


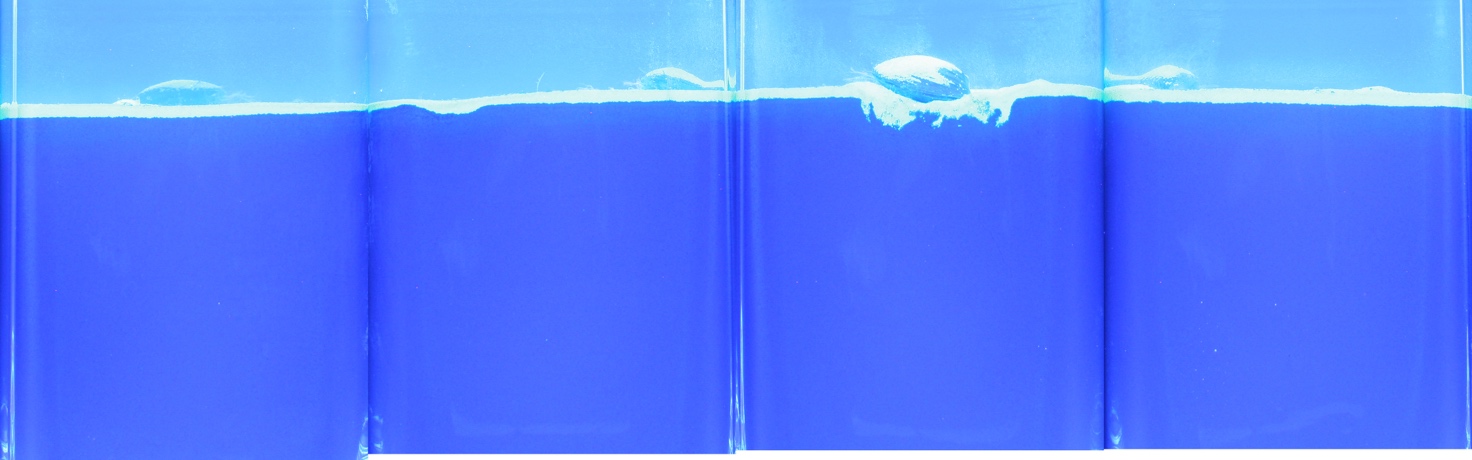


Figure S4(c)


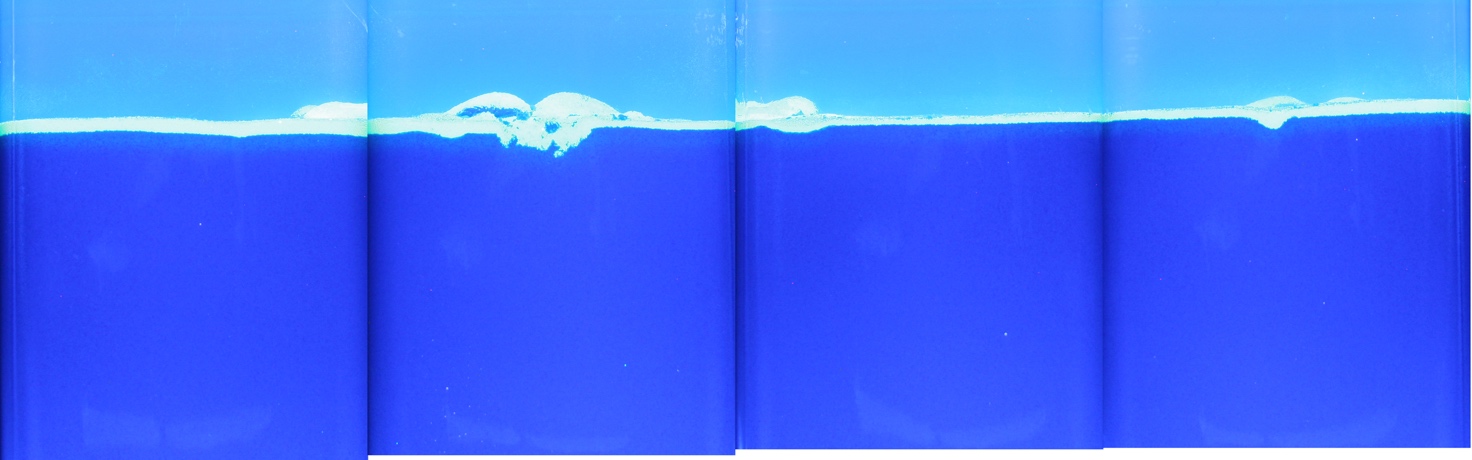


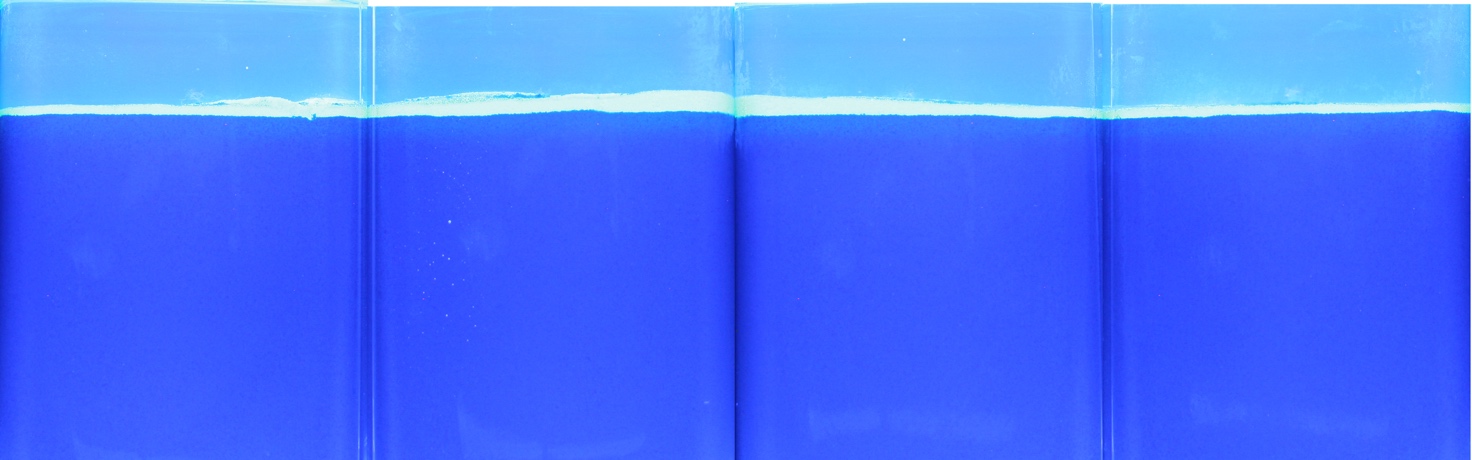


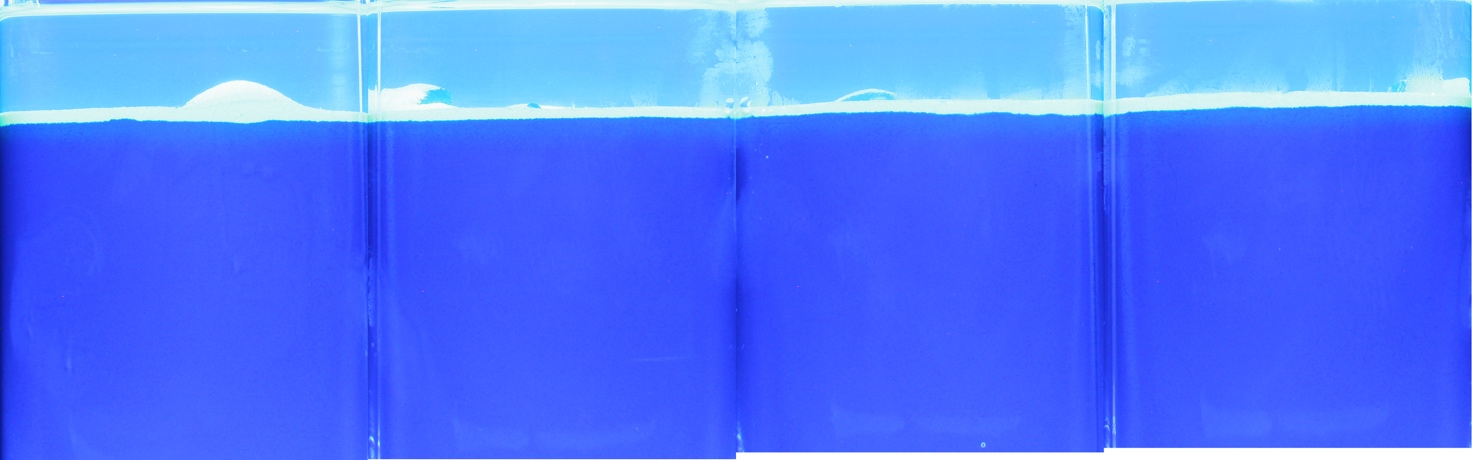


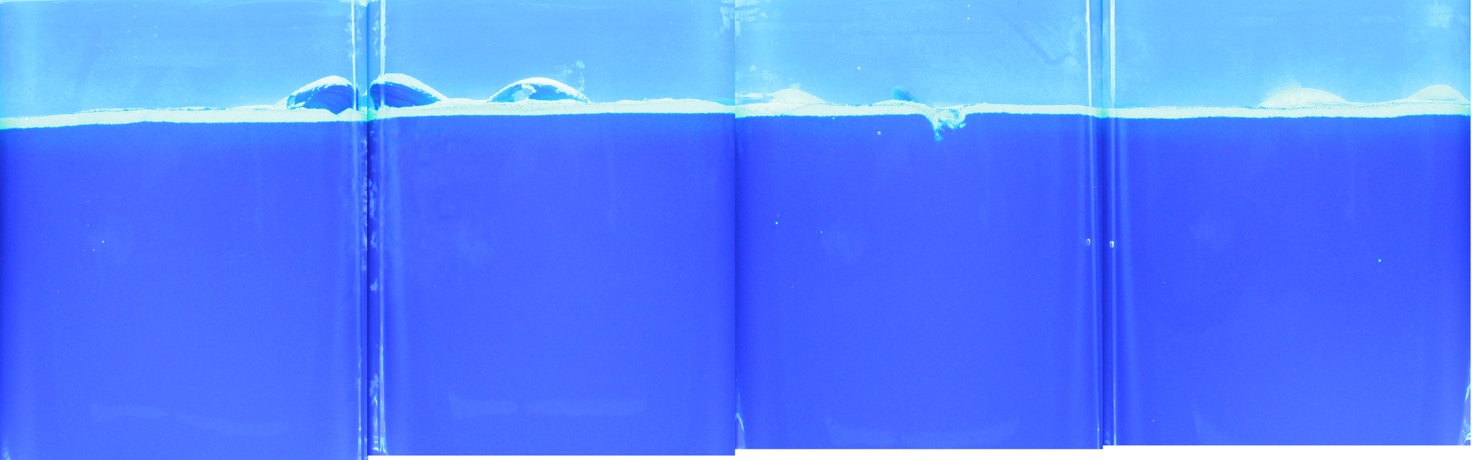


Figure S4(d)


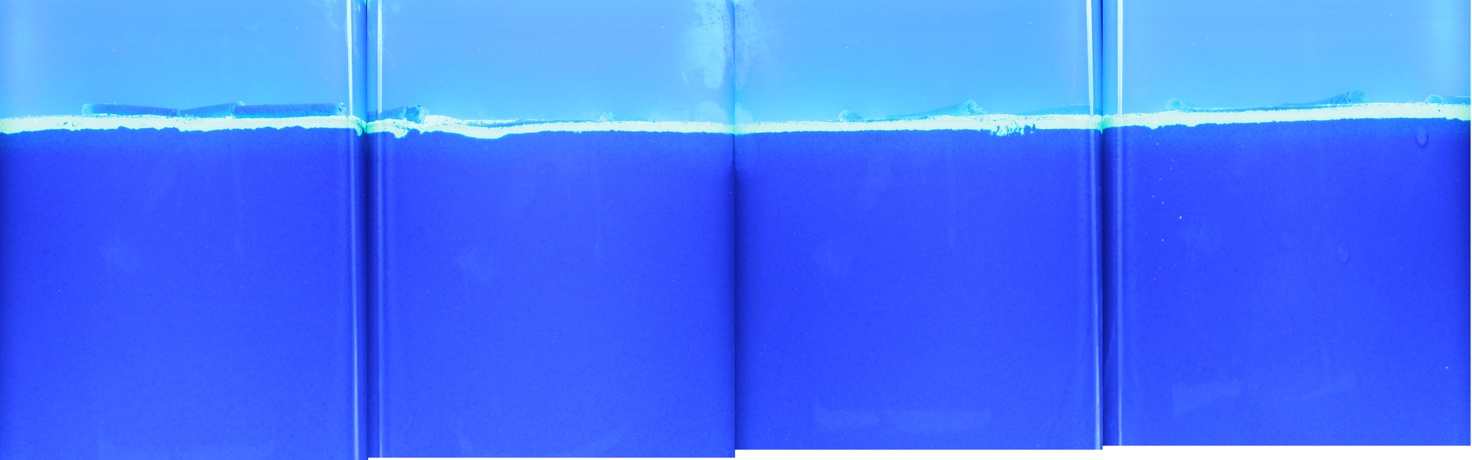


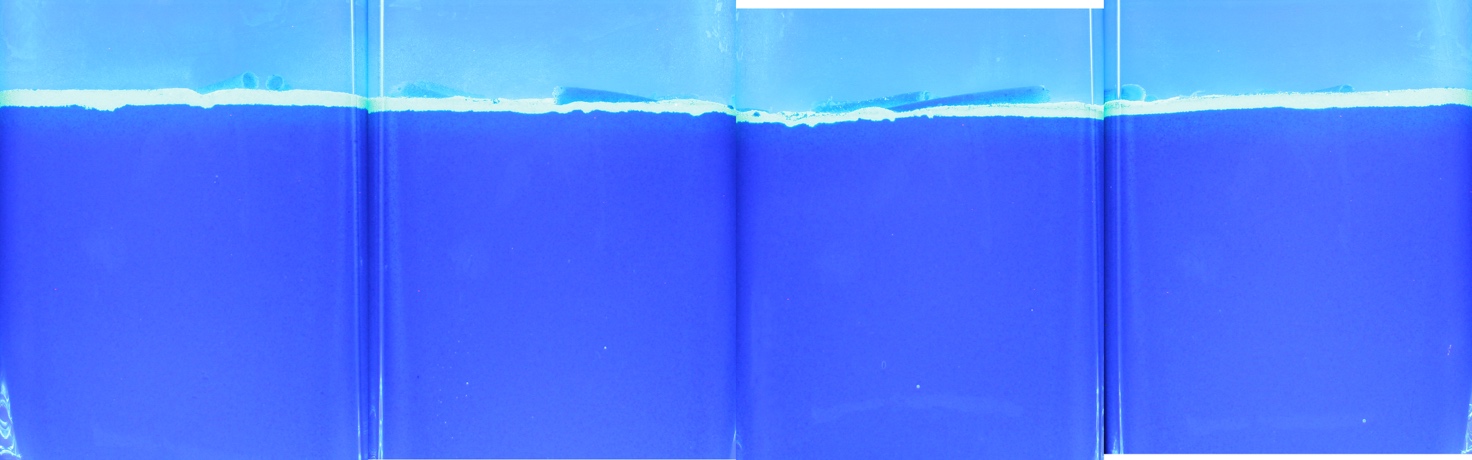


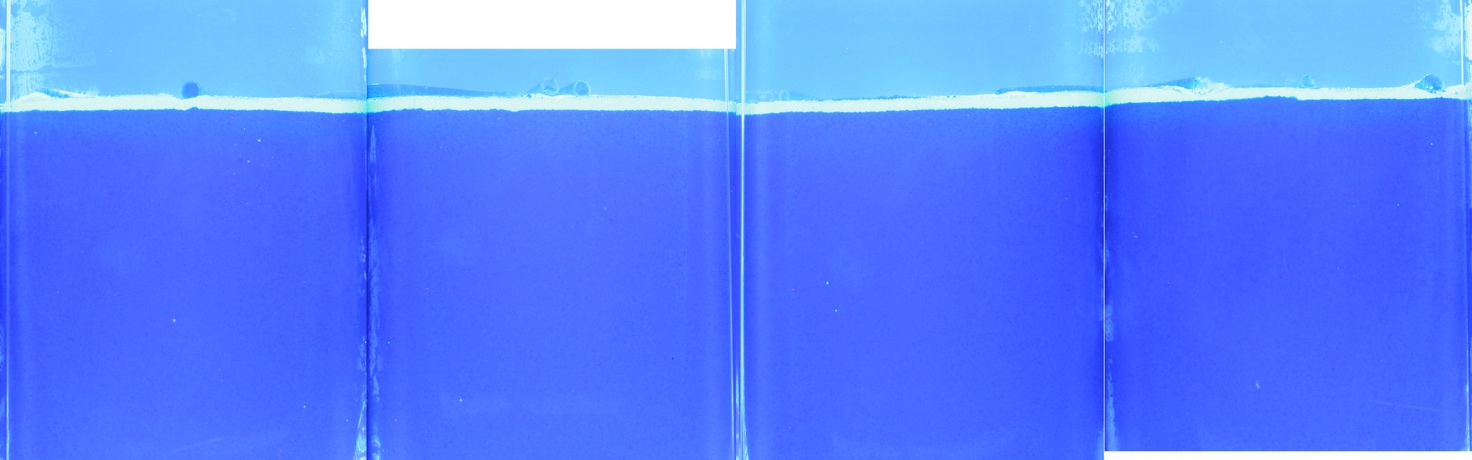


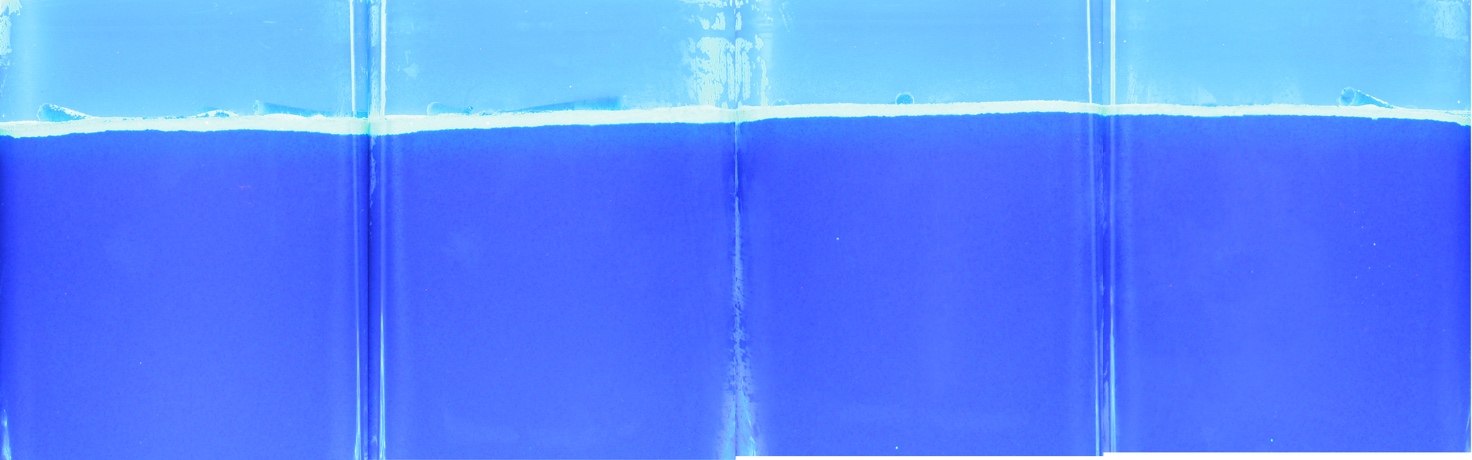


Figure S4(e)


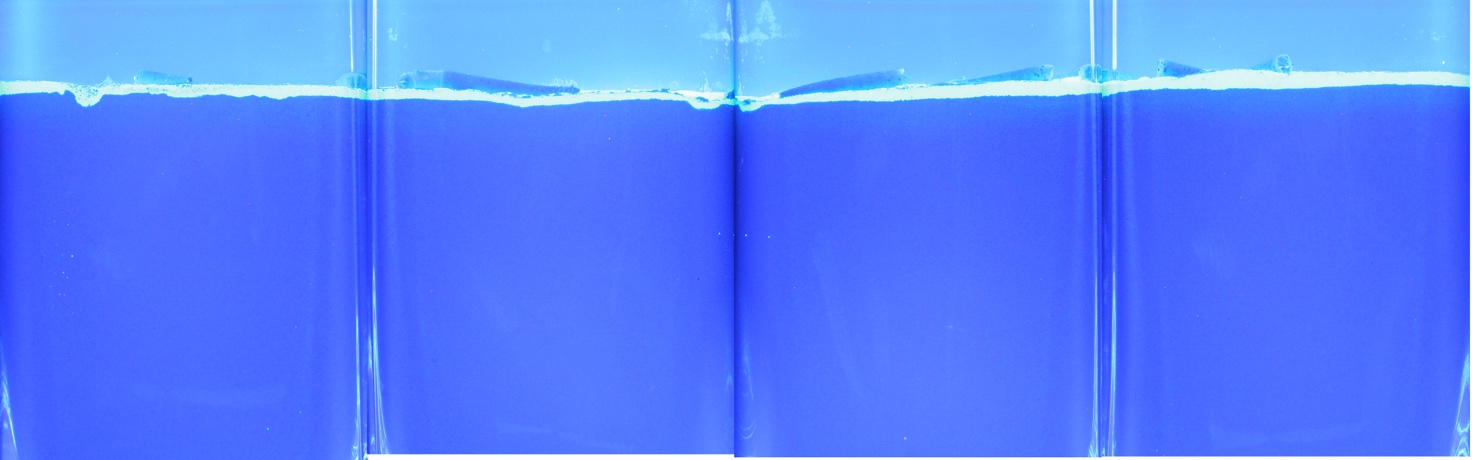


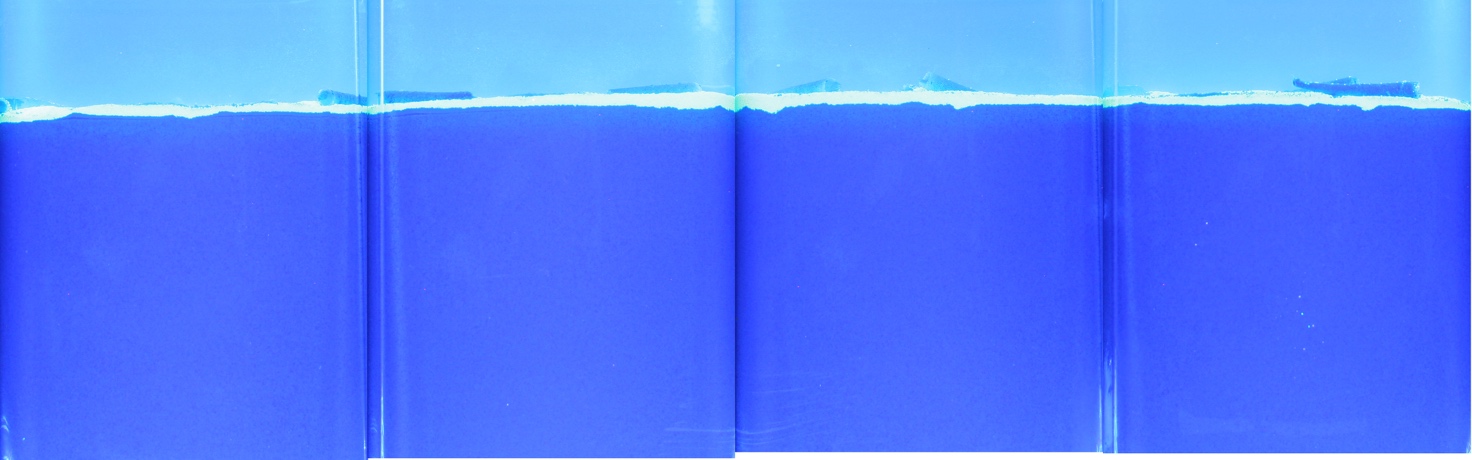


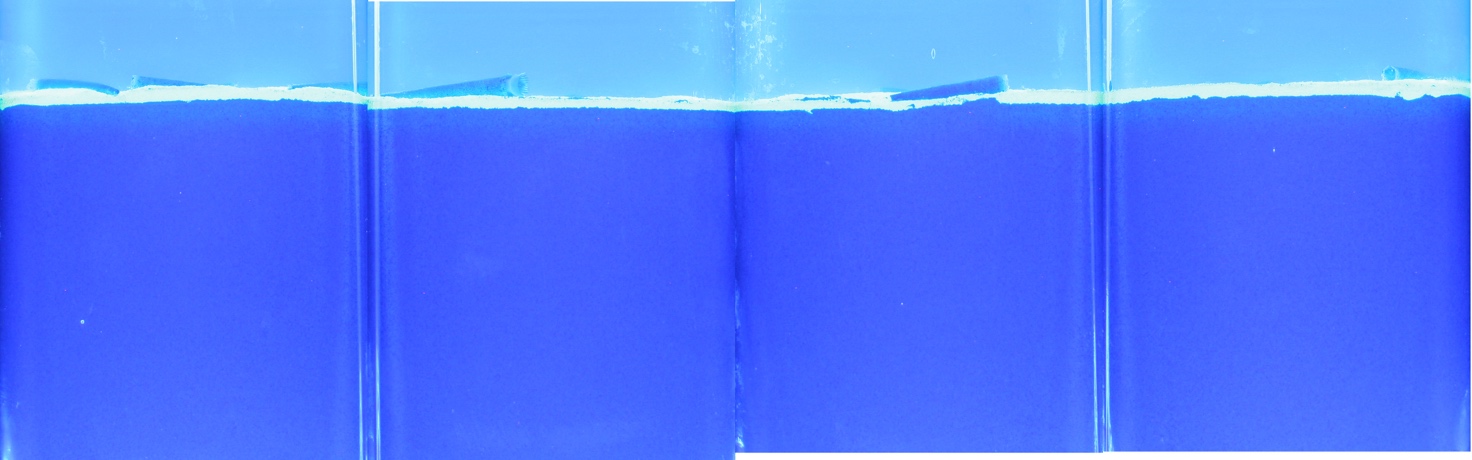


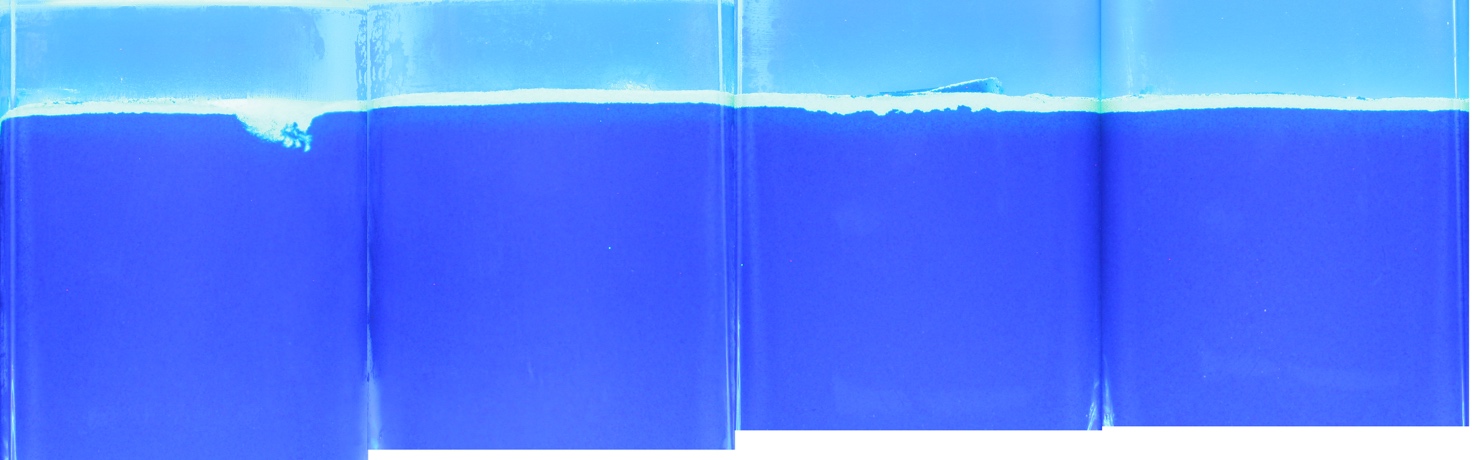


Figure S4(f)


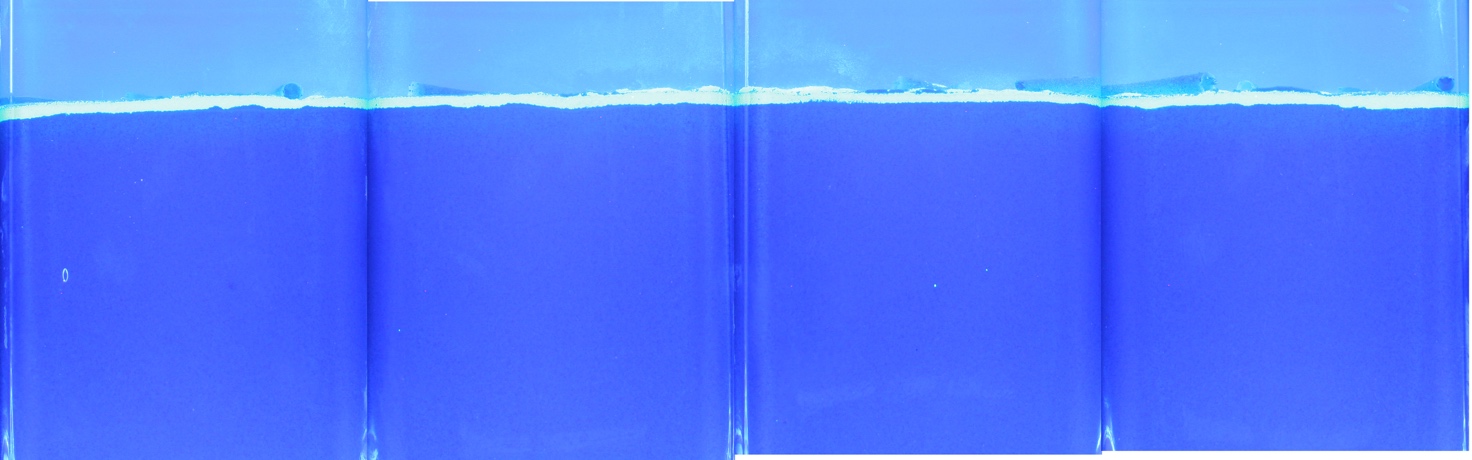


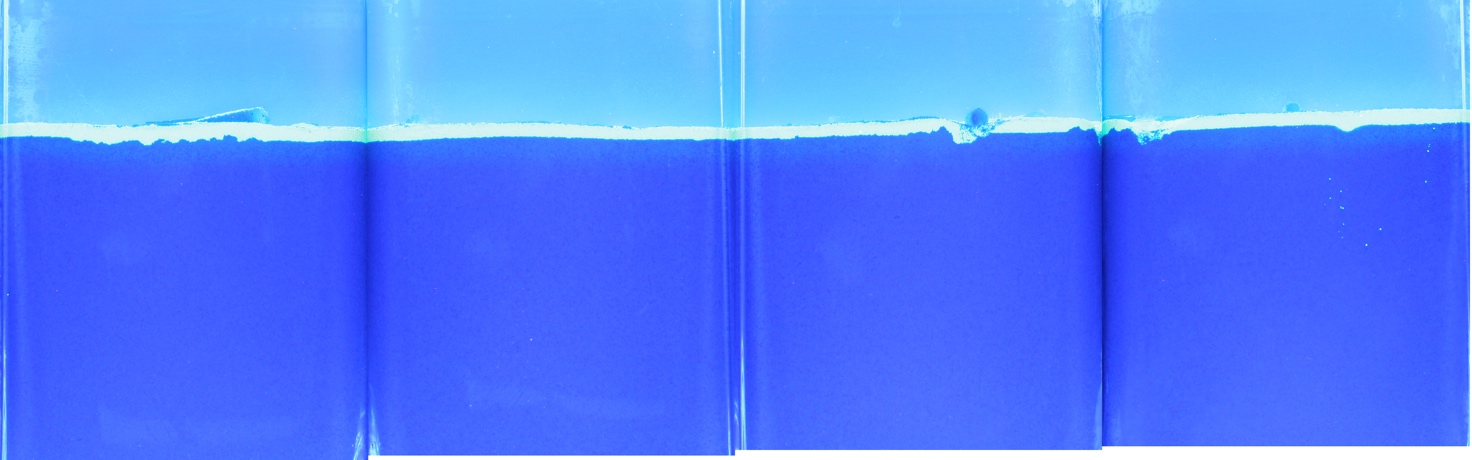


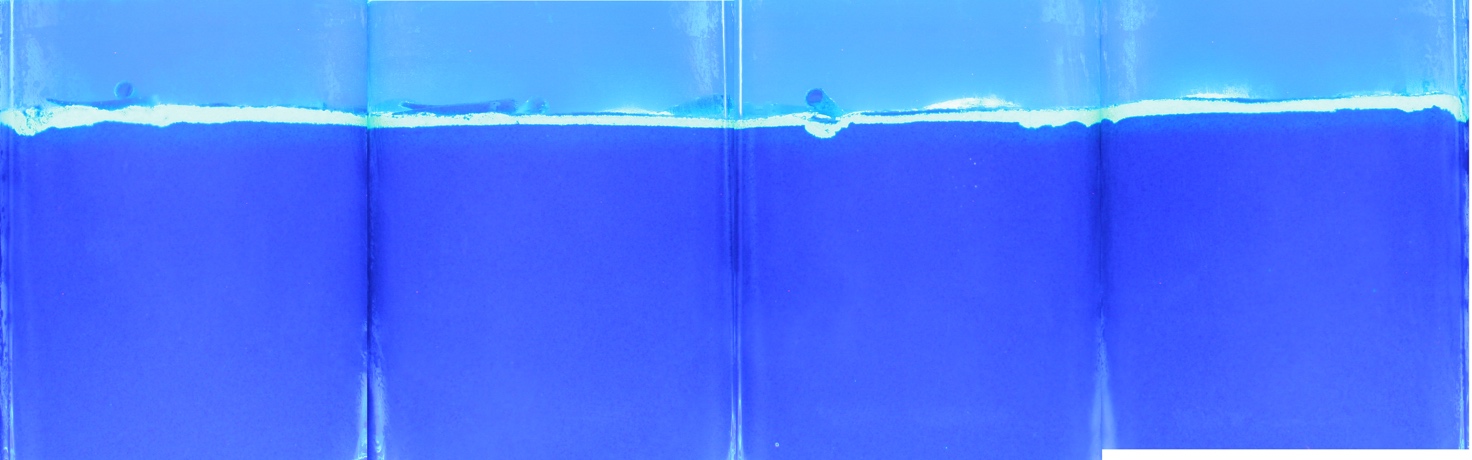


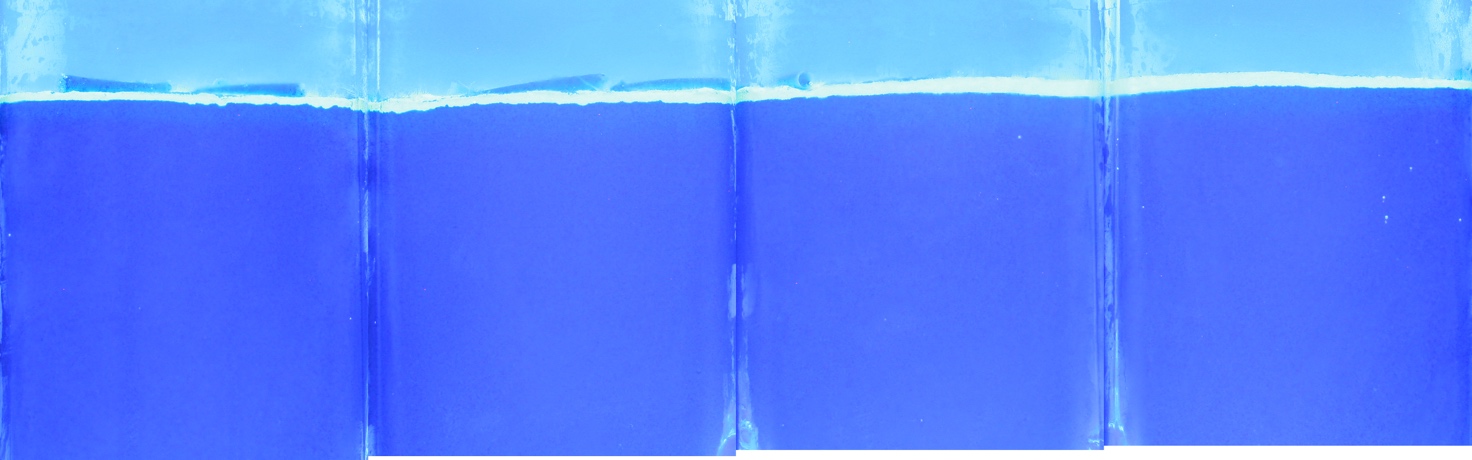


Figure S4(g)


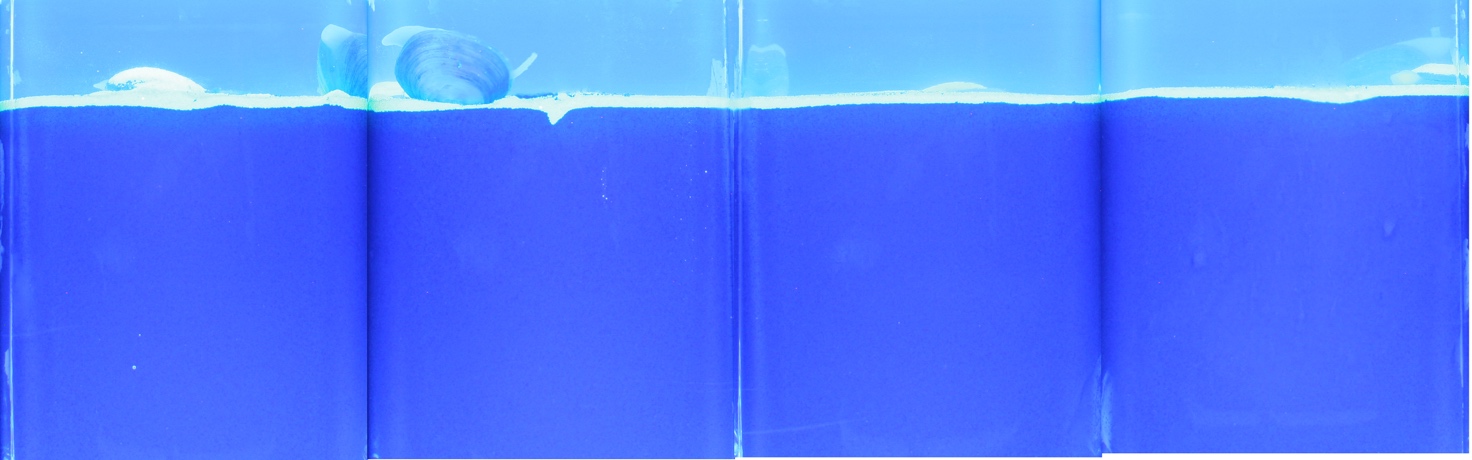


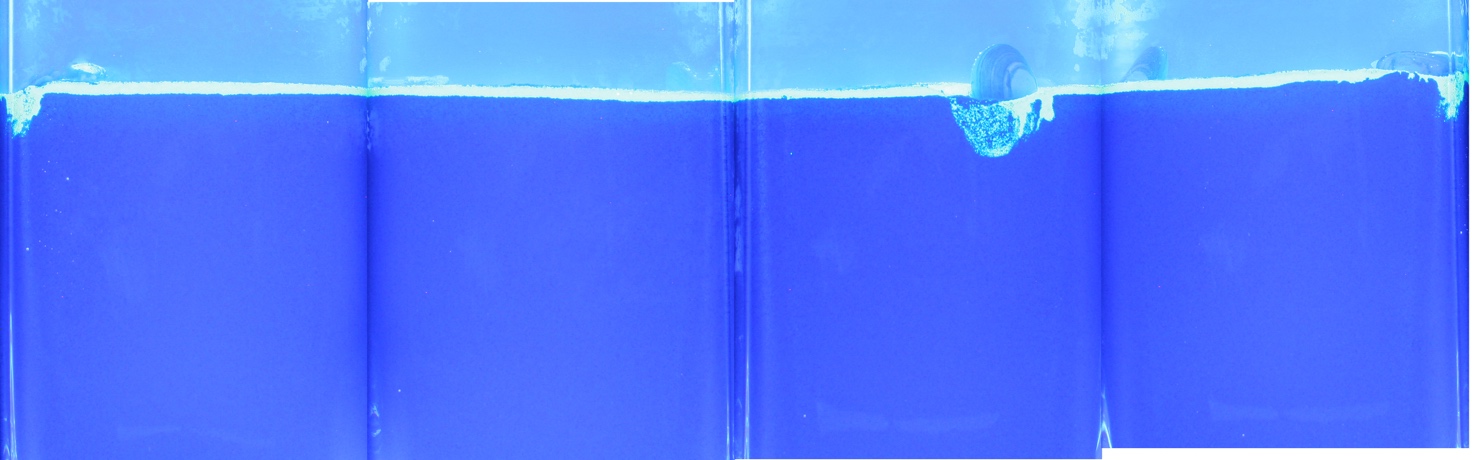


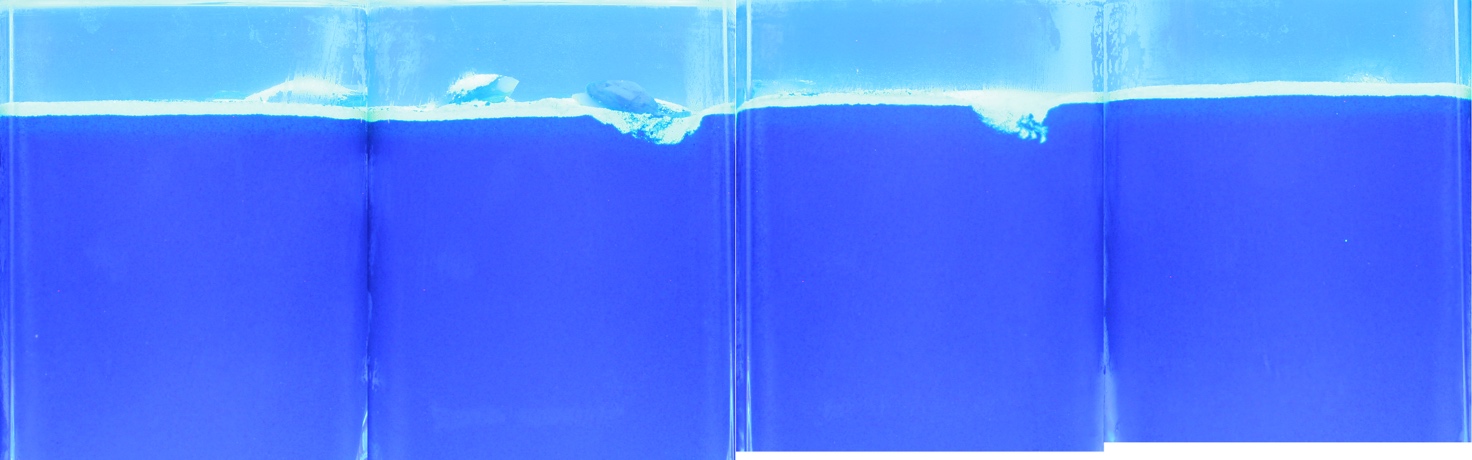


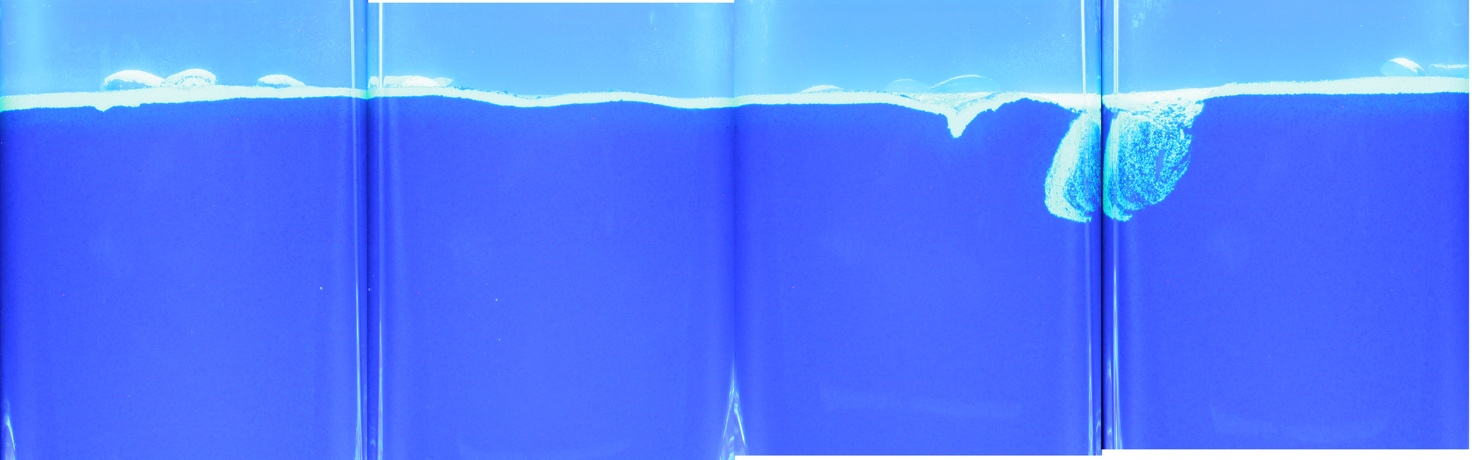


Figure S4(h)


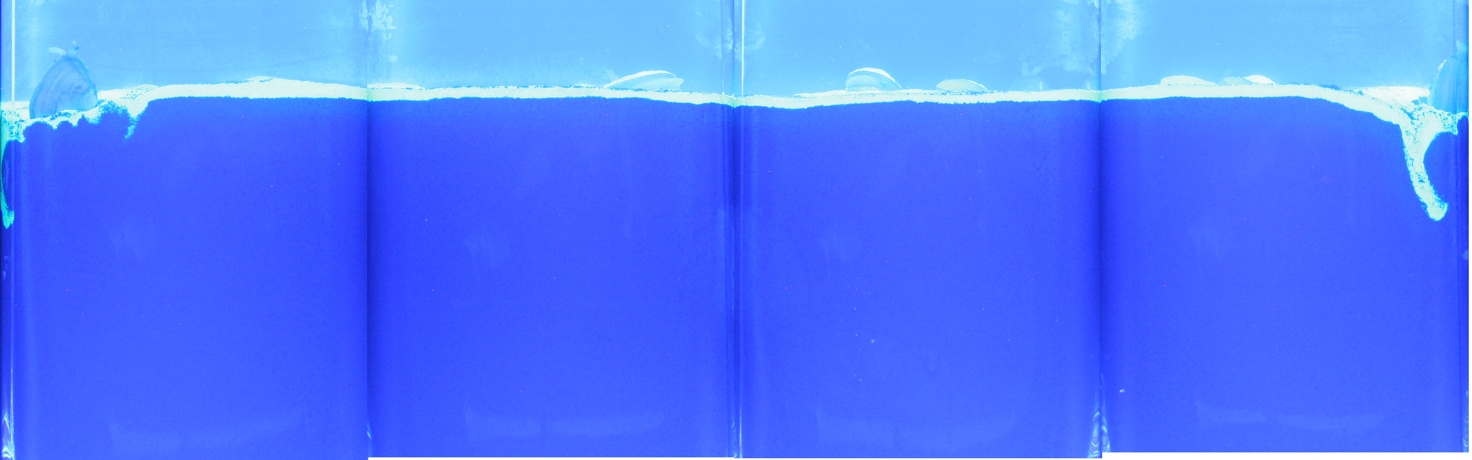


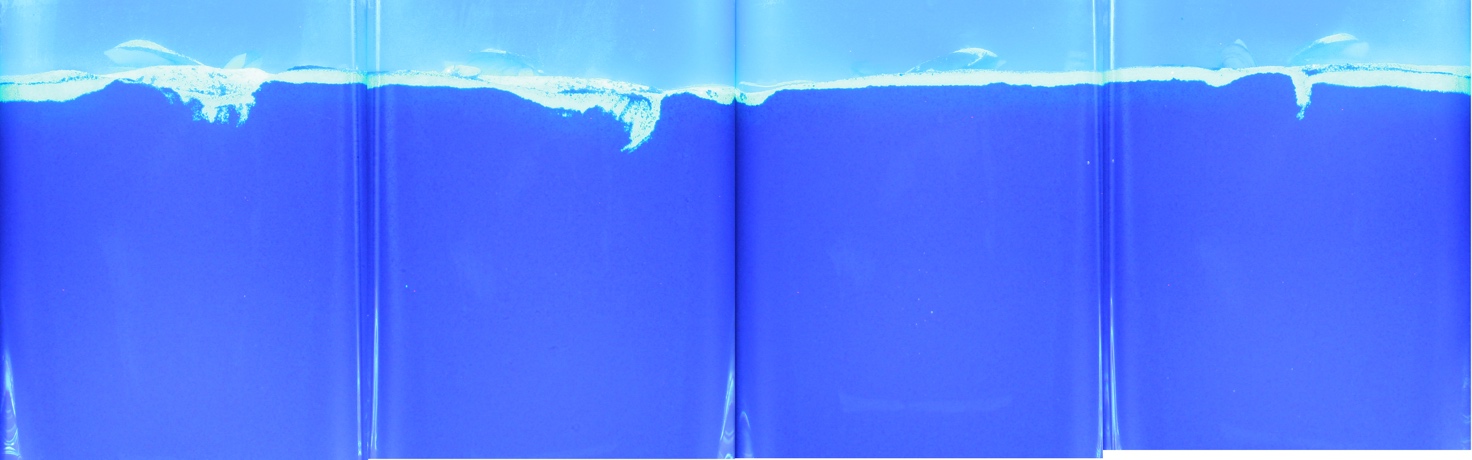


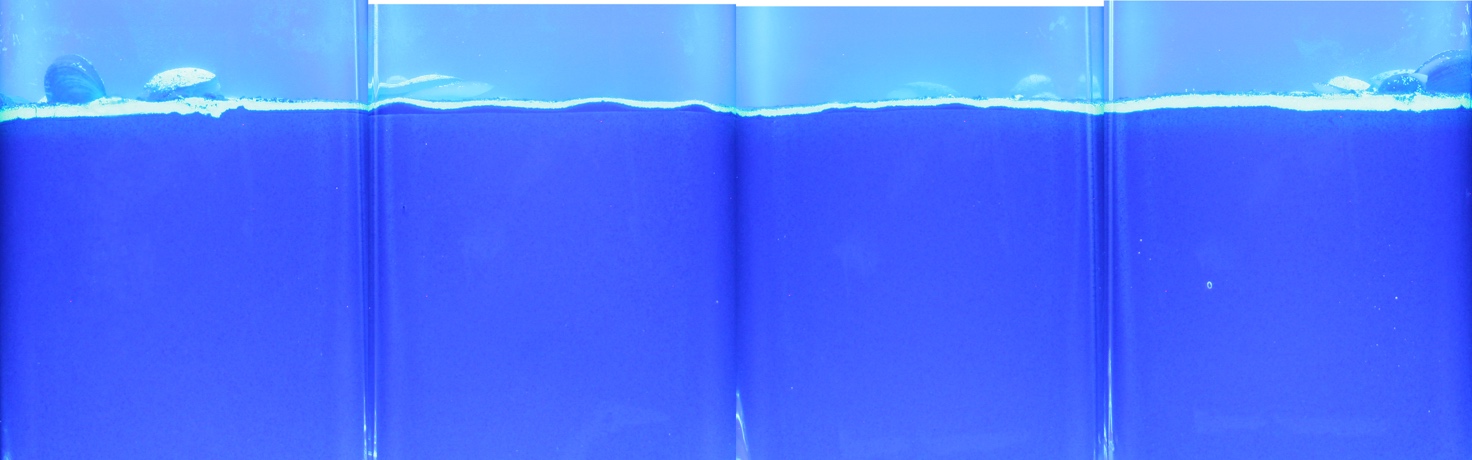


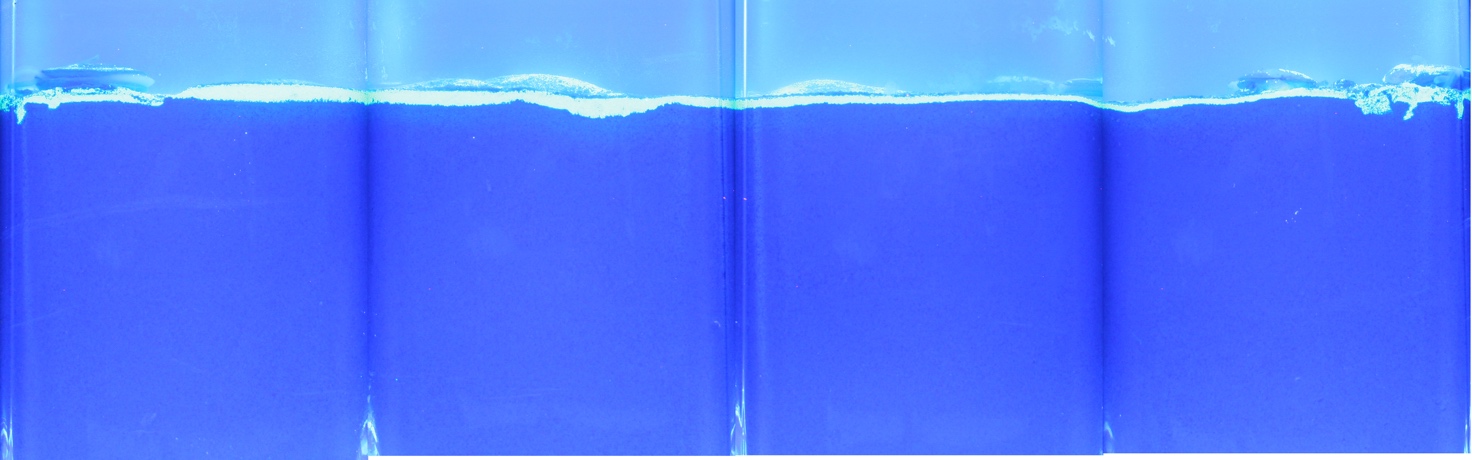


Figure S4(i)


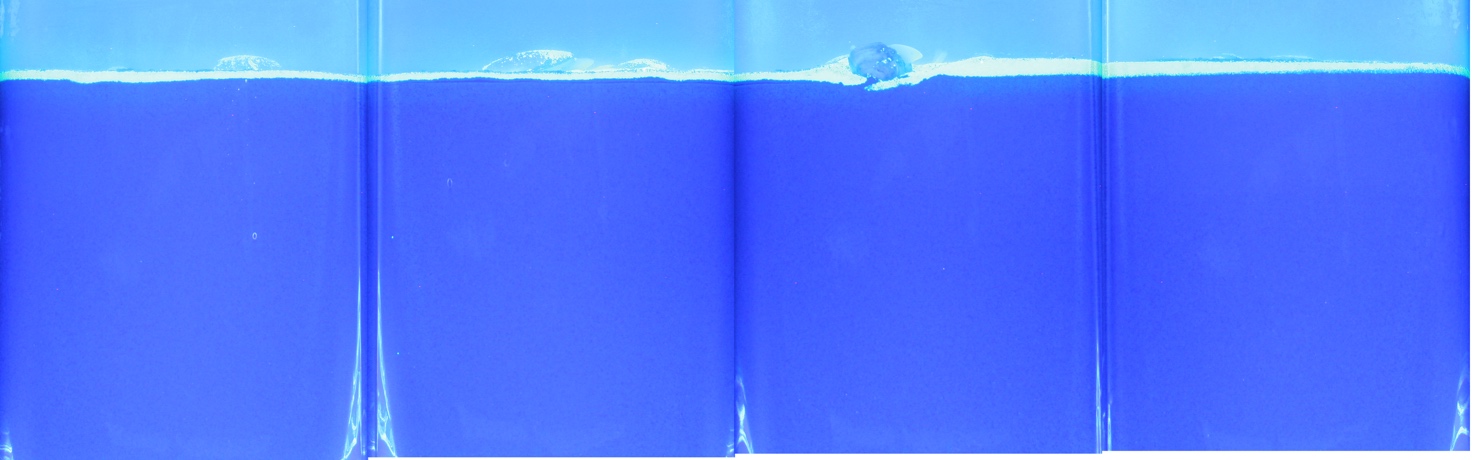


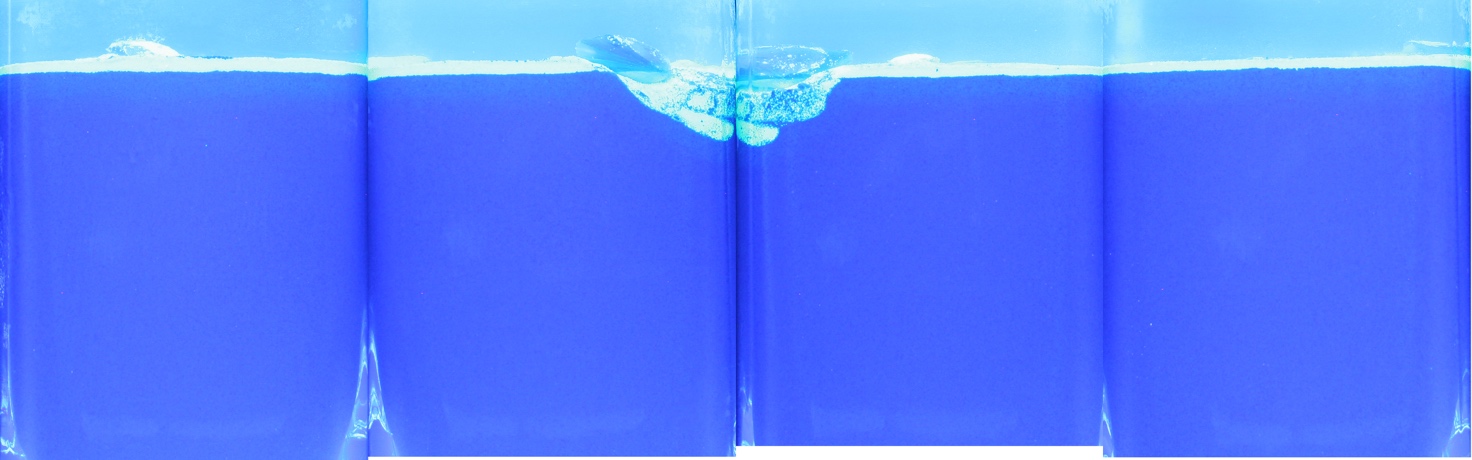


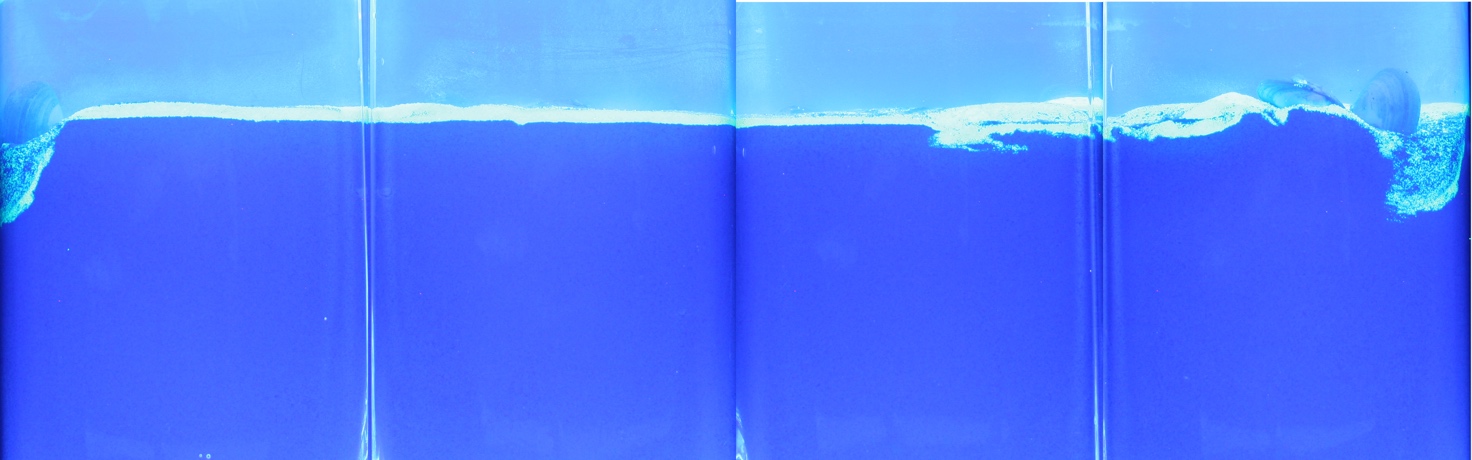


**Figure S5 |** Sediment profiles per aquaria containing (a-c) *Astarte crenata* (d-f) *Cistenides hyperborea* and (g-i) *Macoma calcarea* in the presence of (a,d,g) 0% (b,e,h) 0.1% and (c,f,i) 0.5% of the water accommodated fraction of marine fuel oil. Dosing concentrations are relative to above-sediment water volume.


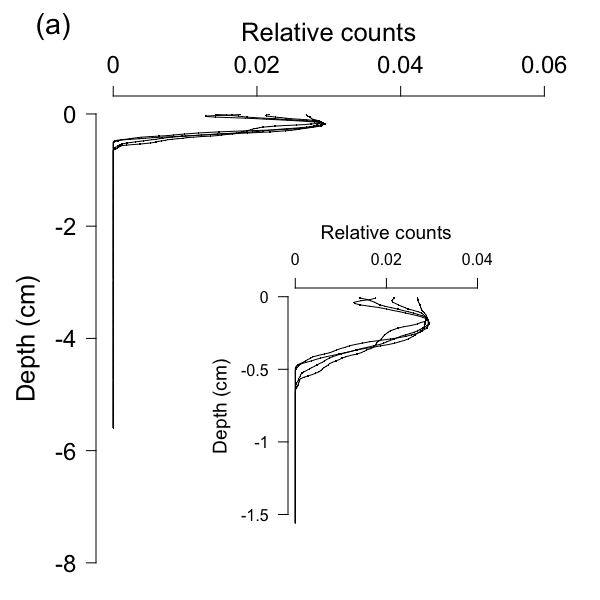

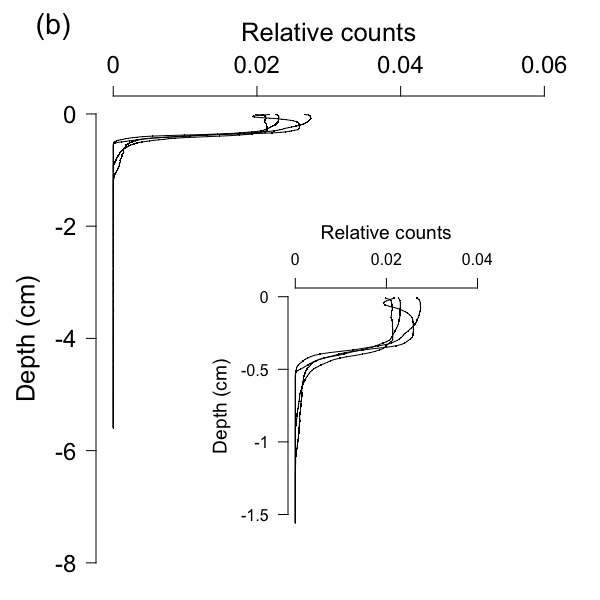

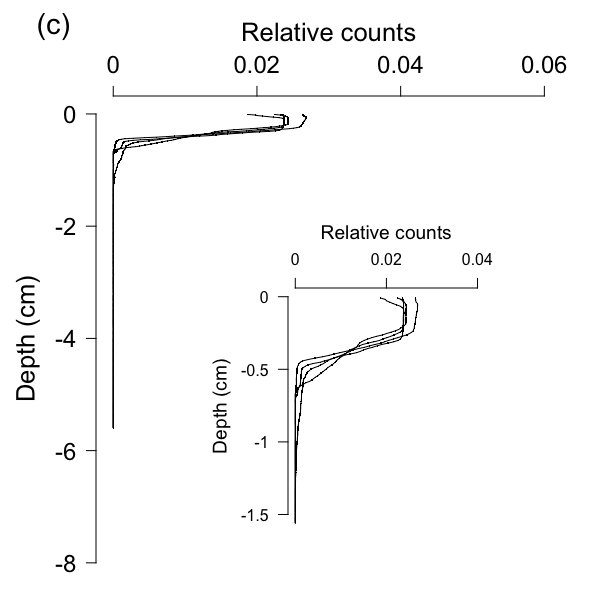

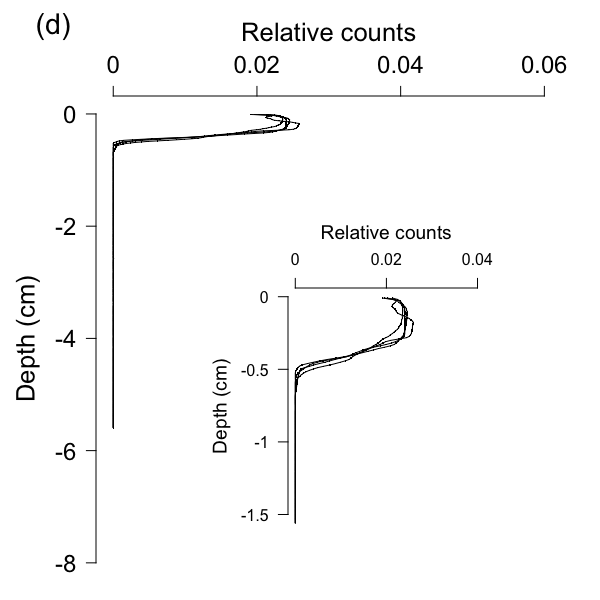

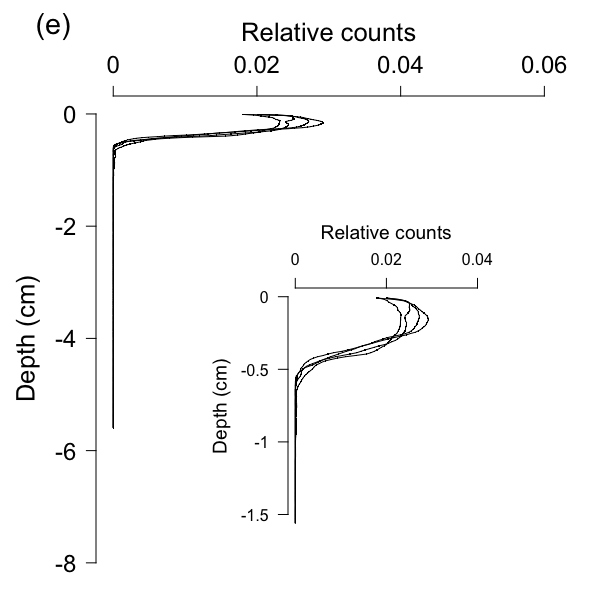

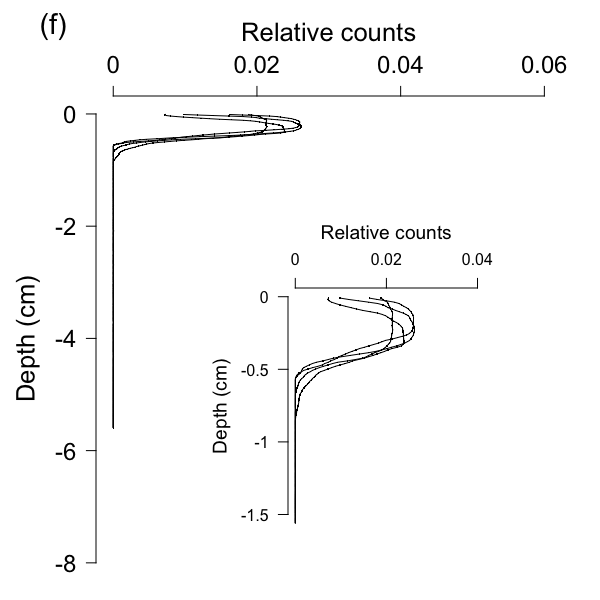

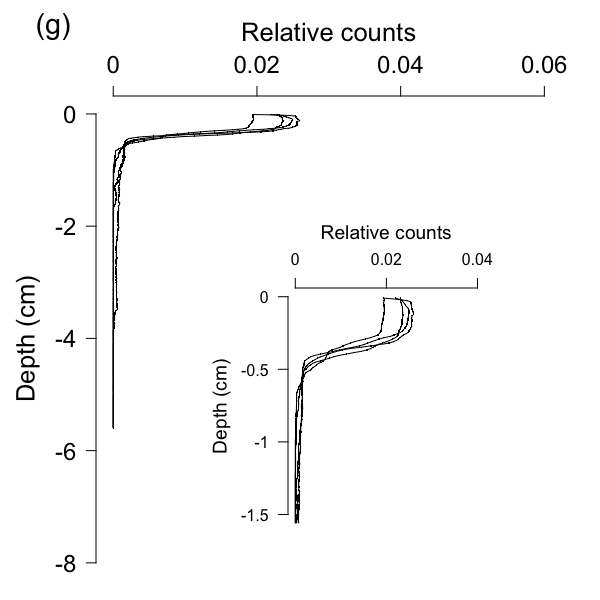

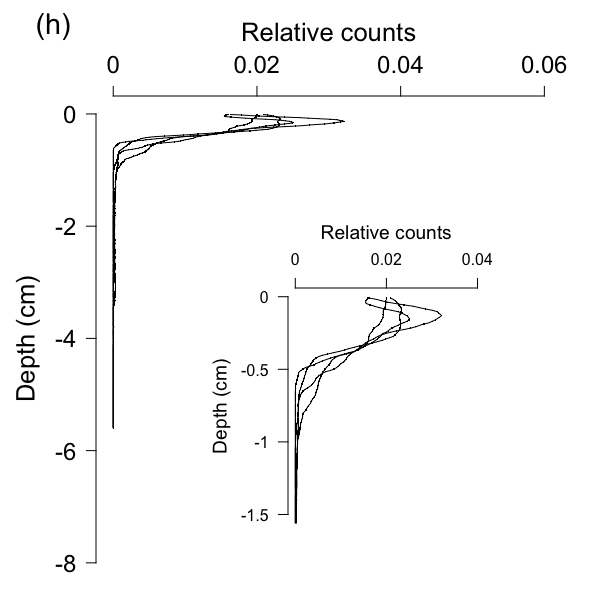

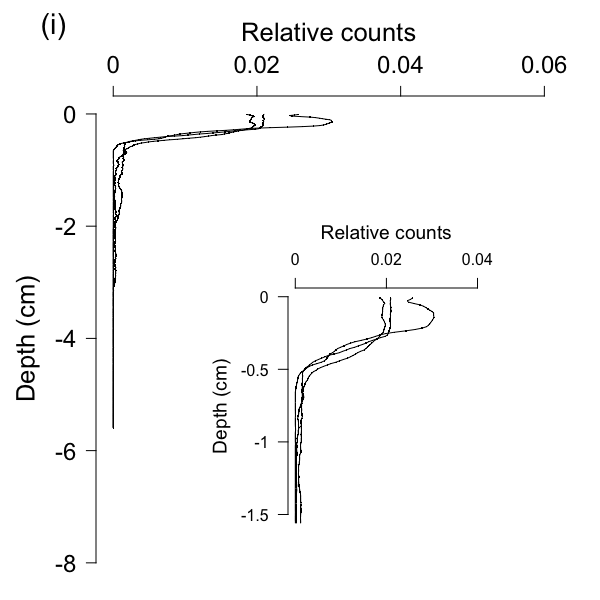


**Table S1** | Summary of sediment particle size statistics for four random mesocosms and sample of luminophores given by the default GRADISTAT output (Blott & Pye, 2001). Mean, sorting, skewness, kurtosis, the percentage of sample less than 63 μm and total organic material are presented for all sediment samples. Superscripts provide descriptive terminology as outlined by Blott & Pye (2001). **Mean, *x̄* :** fs, fine sand; ms, medium sand; cs, coarse sand; vcs, very coarse sand. **Sorting, *σ*:** ps, poorly sorted; vps, very poorly sorted. **Skewness, *Sk*:** sy, symmetrical; vfsk, very fine skewed; fsk, fine skewed; csk, coarse skewed. **Kurtosis, *K*:** mk, mesokurtic; lk, leptokurtic; pk, platykurtic. Organic matter content was determined by loss on ignition.

| **Sample** | **Mean (x̄ , μm)** | **Sorting (σ, μm)** | **Skewness (Sk, μm)** | **Kurtosis (K, μm)** | **Sample <63 μm (%)** | **Organic material (%)** | **Sediment name** |  |
| --- | --- | --- | --- | --- | --- | --- | --- | --- |
|  |  |  |  |  |  |  |  |  |
| ***Sediment*** |  |  |  |  |  |  |  |  |
| *1* | 136^fs^ | 228.51^ps^ | 1.75^sy^ | 6.56^pk^ | 23.49 | 0.11 | Poorly Sorted Very Fine Sand |  |
| *2* | 160^fs^ | 239.34^ps^ | 2.07^sy^ | 8.99^pk^ | 16.62 | 0.44 | Slightly Very Fine Gravelly Medium Sand |  |
| *3* | 178^fs^ | 248.04^ps^ | 3.69^fsk^ | 34.07^pk^ | 15.15 | 0.30 | Slightly Very Fine Gravelly Medium Sand |  |
| *4* | 127^fs^ | 196.00^ps^ | 1.99^sy^ | 8.11^pk^ | 22.17 | 0.34 | Poorly Sorted Very Fine Sand |  |
|  |  |  |  |  |  |  |  |  |
| ***Luminophores*** |  |  |  |  |  |  |  |  |
| *1* | 301^ms^ | 124.75^ws^ | 1.13^sy^ | 4.85^mk^ | 0 | N/A | Well Sorted Medium Sand |  |

**Statistical model summary**

Each species (*A. crenata*, *M. calcerea*, *C. hyperborea*) was analysed separately (Model S1 to S12). For each model we list the initial linear regression model and the minimal adequate model. When homogeneity of variance was violated, we used a linear regression with generalised least squares (GLS) estimation. We present a summary of the coefficient tables for single terms. The coefficients indicate the relative performance of each factor level in relation to the re-levelled baseline (as indicated). Coefficients ± SE, t-values and respective significance values are presented.

**Abbreviations**

1. **Explanatory variables**

Oil, Marine fuel oil concentration (%, water accommodated fraction relative to above-sediment water volume).

1. **Response variables**

SBR, surface boundary roughness (cm)

^f-SPI^L_mean_, mean mixed depth of particle reworking (cm)

^f-SPI^L_median_, median mixed depth of particle reworking (cm)

^f-SPI^L_max_ , maximum mixed depth of particle reworking (cm)

**Data:** All data used in the analyses are provided as Table S2.

***Astarte crenata***

**Model S1** Surface boundary roughness (SBR, cm)

Initial linear regression model:

lm(SBR ~ as.factor(Oil))

Minimal adequate model:

gls(SBR ~ 1,

weights = varIdent(form = ~ 1|as.factor(Oil), method = "REML")

**Model S2** Mean mixing depth (^f-SPI^L_mean_, cm)

Initial linear regression model:

lm(^f-SPI^L_mean_ ~ as.factor(Oil))

Minimal adequate model:

gls(^f-SPI^L_mean_ ~ as.factor(Oil),

weights = varIdent(form = ~ 1|as.factor(Oil), method = "REML")

Intercept ± SE (when baseline is for 0 % for marine fuel oil concentration): 0.35 ± 0.01, t = 32.24, **p < 0.0001**

Coefficient table for Oil

|  | 0 | 0.1 | 0.5 |
| --- | --- | --- | --- |
| 0 | / | 0.07 ± 0.03  2.67  **0.026** | 0.05 ± 0.01  4.40  **0.002** |
| 0.1 | 0.07 ± 0.03  2.67  **0.026** | / | -0.01 ± 0.02  -0.06  0.597 |
| 0.5 | 0.05 ± 0.01  4.40  **0.002** | -0.01 ± 0.02  -0.06  0.597 | / |

**Model S3** Median mixing depth (^f-SPI^L_median_, cm)

Initial linear regression model:

lm(^f-SPI^L_median_ ~ as.factor(Oil))

Minimal adequate model:

gls(^f-SPI^L_median_ ~ 1,

weights = varIdent(form = ~ 1|as.factor(Oil), method = "REML")

**Model S4** Max mixing depth (^f-SPI^L_max_, cm)

Initial linear regression model:

lm(^f-SPI^L_max_ ~ as.factor(Oil))

Minimal adequate model:

gls(^f-SPI^L_max_ ~ as.factor(Oil),

weights = varIdent(form = ~ 1|as.factor(Oil), method = "REML")

Intercept ± SE (when baseline is for 0 % for marine fuel oil concentration): 0.58 ± 0.04, t = 13.18, **p < 0.0001**

Coefficient table for Oil

|  | 0 | 0.1 | 0.5 |
| --- | --- | --- | --- |
| 0 | / | 0.22 ± 0.16  1.37  0.205 | 0.35 ± 0.16  2.19  **0.056** |
| 0.1 | 0.22 ± 0.16  1.37  0.205 | / | 0.13 ± 0.22  0.58  0.574 |
| 0.5 | 0.35 ± 0.16  2.19  **0.056** | 0.13 ± 0.22  0.58  0.574 | / |

***Cistenides hyperborea***

**Model S5** Surface boundary roughness (SBR, cm)

Initial linear regression model:

lm(SBR ~ as.factor(Oil))

Minimal adequate model:

gls(SBR ~ 1,

weights = varIdent(form = ~ 1|as.factor(Oil), method = "REML")

**Model S6** Mean mixing depth (^f-SPI^L_mean_, cm)

Initial linear regression model:

lm(^f-SPI^L_mean_ ~ as.factor(Oil))

Minimal adequate model:

gls(^f-SPI^L_mean_ ~ as.factor(Oil),

weights = varIdent(form = ~ 1|as.factor(Oil), method = "REML")

Intercept ± SE (when baseline is for 0 % for marine fuel oil concentration): 0.41 ± 0.01, t = 48.57, **p < 0.0001**

Coefficient table for Oil

|  | 0 | 0.1 | 0.5 |
| --- | --- | --- | --- |
| 0 | / | -0.03 ± 0.02  -1.52  0.162 | 0.04 ± 0.03  1.60  0.144 |
| 0.1 | -0.03 ± 0.02  -1.52  0.162 | / | 0.07 ± 0.03  2.36  **0.042** |
| 0.5 | 0.04 ± 0.03  1.60  0.144 | 0.07 ± 0.03  2.36  **0.042** | / |

**Model S7** Median mixing depth (^f-SPI^L_median_, cm)

Initial linear regression model:

lm(^f-SPI^L_median_ ~ as.factor(Oil))\

Minimal adequate model:

gls(^f-SPI^L_median_ ~ 1,

weights = varIdent(form = ~ 1|as.factor(Oil), method = "REML")

**Model S8** Max mixing depth (^f-SPI^L_max_, cm)

Initial linear regression model:

lm(^f-SPI^L_max_ ~ as.factor(Oil))

Minimal adequate model:

gls(^f-SPI^L_max_ ~ 1,

weights = varIdent(form = ~ 1|as.factor(Oil), method = "REML")

***Macoma calcerea***

**Model S9** Surface boundary roughness (SBR, cm)

Initial linear regression model:

lm(SBR ~ as.factor(Oil))

Minimal adequate model:

gls(SBR ~ 1,

weights = varIdent(form = ~ 1|as.factor(Oil), method = "REML")

**Model S10** Mean mixing depth (^f-SPI^L_mean_, cm)

Initial linear regression model:

lm(^f-SPI^L_mean_ ~ as.factor(Oil))

Minimal adequate model:

gls(^f-SPI^L_mean_ ~ 1,

weights = varIdent(form = ~ 1|as.factor(Oil), method = "REML")

**Model S11** Median mixing depth (^f-SPI^L_median_, cm)

Initial linear regression model:

lm(^f-SPI^L_median_ ~ as.factor(Oil))

Minimal adequate model:

gls(^f-SPI^L_median_ ~ 1,

weights = varIdent(form = ~ 1|as.factor(Oil), method = "REML")

**Model S12** Max mixing depth (^f-SPI^L_max_, cm)

Initial linear regression model:

lm(^f-SPI^L_max_ ~ as.factor(Oil))

Minimal adequate model:

gls(^f-SPI^L_max_ ~ 1,

weights = varIdent(form = ~ 1|as.factor(Oil), method = "REML")

**Table S2 |** Summary of bioturbation indicators used in our statistical analyses. Oil = marine fuel oil (%, water accommodated fraction relative to above-sediment water volume). * indicates loss of aquarium in error.

| **Species identity** | **Oil** | **Total biomass (g)** | **SBR (cm)** | **^f-SPI^L_median_ (cm)** | **^f-SPI^L_mean_ (cm)** | **^f-SPI^L_max_ (cm)** |
| --- | --- | --- | --- | --- | --- | --- |
| Astarte crenata | 0 | 11.4 | 0.622 | 0.323 | 0.311 | 0.499 |
| Astarte crenata | 0 | 10.9 | 1.211 | 0.335 | 0.338 | 0.516 |
| Astarte crenata | 0 | 12.1 | 0.481 | 0.366 | 0.358 | 0.650 |
| Astarte crenata | 0 | 10.6 | 1.038 | 0.365 | 0.349 | 0.670 |
| Astarte crenata | 0.1 | 10.7 | 0.751 | 0.439 | 0.404 | 0.901 |
| Astarte crenata | 0.1 | 13.7 | 0.563 | 0.386 | 0.356 | 0.553 |
| Astarte crenata | 0.1 | 11.7 | 0.647 | 0.368 | 0.347 | 0.563 |
| Astarte crenata | 0.1 | 11 | 0.687 | 0.468 | 0.376 | 1.204 |
| Astarte crenata | 0.5 | 11.7 | 0.751 | 0.412 | 0.366 | 1.334 |
| Astarte crenata | 0.5 | 13.6 | 0.546 | 0.412 | 0.358 | 0.678 |
| Astarte crenata | 0.5 | 10.2 | 0.714 | 0.399 | 0.385 | 0.714 |
| Astarte crenata | 0.5 | 11.7 | 0.582 | 0.386 | 0.347 | 1.004 |
| Cistenides hyperborea | 0 | 2.8 | 0.667 | 0.403 | 0.376 | 0.695 |
| Cistenides hyperborea | 0 | 3.59 | 1.025 | 0.429 | 0.423 | 0.790 |
| Cistenides hyperborea | 0 | 3.2 | 0.461 | 0.390 | 0.386 | 0.593 |
| Cistenides hyperborea | 0 | 3.4 | 0.678 | 0.399 | 0.395 | 0.602 |
| Cistenides hyperborea | 0.1 | 3.41 | 0.989 | 0.370 | 0.339 | 0.640 |
| Cistenides hyperborea | 0.1 | 2.93 | 0.677 | 0.345 | 0.329 | 0.602 |
| Cistenides hyperborea | 0.1 | 3.1 | 0.490 | 0.381 | 0.377 | 0.640 |
| Cistenides hyperborea | 0.1 | 2.7 | 0.385 | 0.419 | 0.395 | 1.128 |
| Cistenides hyperborea | 0.5 | 3.3 | 0.464 | 0.415 | 0.388 | 0.577 |
| Cistenides hyperborea | 0.5 | 2.9 | 0.581 | 0.505 | 0.431 | 0.722 |
| Cistenides hyperborea | 0.5 | 3 | 0.715 | 0.461 | 0.432 | 0.921 |
| Cistenides hyperborea | 0.5 | 2.8 | 0.928 | 0.400 | 0.356 | 0.572 |
| Macoma calcarea | 0 | 9.2 | 0.481 | 0.386 | 0.340 | 0.991 |
| Macoma calcarea | 0 | 5.6 | 0.801 | 0.503 | 0.339 | 1.686 |
| Macoma calcarea | 0 | 6.6 | 1.108 | 0.450 | 0.376 | 1.221 |
| Macoma calcarea | 0 | 6.5 | 0.668 | 0.675 | 0.339 | 3.896 |
| Macoma calcarea | 0.1 | 7.3 | 1.148 | 0.471 | 0.348 | 3.453 |
| Macoma calcarea | 0.1 | 7.4 | 0.932 | 0.508 | 0.386 | 1.835 |
| Macoma calcarea | 0.1 | 9 | 0.562 | 0.350 | 0.290 | 0.655 |
| Macoma calcarea | 0.1 | 7.9 | 0.658 | 0.465 | 0.366 | 1.052 |
| Macoma calcarea | 0.5 | 7.5 | 0.591 | 0.365 | 0.282 | 0.657 |
| Macoma calcarea | 0.5 | 8.3 | 1.091 | 0.521 | 0.367 | 2.059 |
| Macoma calcarea | 0.5 | 7.2 | * | * | * | * |
| Macoma calcarea | 0.5 | 7.6 | 1.235 | 0.603 | 0.440 | 3.171 |

**References**

Blott, S. J., & Pye, K. (2001). GRADISTAT: a grain size distribution and statistics package for the analysis of unconsolidated sediments. *Earth Surface Processes and Landforms, 26*(11), 1237-1248. doi:10.1002/esp.261.

**Ends.**
